# Proteomic and metabolomic analysis of the carotenogenic yeast *Xanthophyllomyces dendrorhous* using different carbon sources

**DOI:** 10.1186/s12864-015-1484-6

**Published:** 2015-04-12

**Authors:** Pilar Martinez-Moya, Karsten Niehaus, Jennifer Alcaíno, Marcelo Baeza, Víctor Cifuentes

**Affiliations:** Departamento de Ciencias Ecológicas, Centro de Biotecnologia, Facultad de Ciencias, Universidad de Chile, Santiago, Chile; Department of Proteome and Metabolome Research, Faculty of Biology, Bielefeld University, Bielefeld, Germany

**Keywords:** Proteomics, Carotenogenesis, Metabolomics, Carbon source, Astaxanthin, ROS

## Abstract

**Background:**

Astaxanthin is a potent antioxidant with increasing biotechnological interest. In *Xanthophyllomyces dendrorhous*, a natural source of this pigment, carotenogenesis is a complex process regulated through several mechanisms, including the carbon source. *X. dendrorhous* produces more astaxanthin when grown on a non-fermentable carbon source, while decreased astaxanthin production is observed in the presence of high glucose concentrations. In the present study, we used a comparative proteomic and metabolomic analysis to characterize the yeast response when cultured in minimal medium supplemented with glucose (fermentable) or succinate (non-fermentable).

**Results:**

A total of 329 proteins were identified from the proteomic profiles, and most of these proteins were associated with carotenogenesis, lipid and carbohydrate metabolism, and redox and stress responses. The metabolite profiles revealed 92 metabolites primarily associated with glycolysis, the tricarboxylic acid cycle, amino acids, organic acids, sugars and phosphates. We determined the abundance of proteins and metabolites of the central pathways of yeast metabolism and examined the influence of these molecules on carotenogenesis.

Similar to previous proteomic-stress response studies, we observed modulation of abundance from several redox, stress response, carbohydrate and lipid enzymes. Additionally, the accumulation of trehalose, absence of key ROS response enzymes, an increased abundance of the metabolites of the pentose phosphate pathway and tricarboxylic acid cycle suggested an association between the accumulation of astaxanthin and oxidative stress in the yeast. Moreover, we observed the increased abundance of late carotenogenesis enzymes during astaxanthin accumulation under succinate growth conditions.

**Conclusions:**

The use of succinate as a carbon source in *X. dendrorhous* cultures increases the availability of acetyl-CoA for the astaxanthin production compared with glucose, likely reflecting the positive regulation of metabolic enzymes of the tricarboxylic acid and glyoxylate cycles. The high metabolite level generated in this pathway could increase the cellular respiration rate, producing reactive oxygen species, which induces carotenogenesis.

**Electronic supplementary material:**

The online version of this article (doi:10.1186/s12864-015-1484-6) contains supplementary material, which is available to authorized users.

## Background

Astaxanthin is a carotenoid with high commercial and biotechnological interest, principally reflecting the antioxidant properties of this organic molecule [1,2]. This pigment has been used in aquaculture, food, and cosmetics, and has been evaluated in the pharmaceutical industry [1,2]. In nature, the microalgae *Haematococcus pluvialis* and the yeast *Xanthophyllomyces dendrorhous* are the primary producers of astaxanthin [1], but the natural production of this pigment does not compete with the chemically synthesized pigment. However, many efforts have intensely focused on increasing astaxanthin production through genetic engineering and the optimization of fermentation conditions [3-6].

*X. dendrorhous* requires the precursor isopentenyl pyrophosphate (IPP), generated via the mevalonate (MVA) pathway, for astaxanthin biosynthesis. Five enzymatic activities are performed in this pathway, and the 3-hydroxy-3-methyl-glutaryl-CoA reductase (HMGR) is a crucial regulator of these processes [7]. Subsequently, IPP is condensed through prenyltransferases with different chain length specificities [8]. Geranylgeranyl pyrophosphate (GGPP) (C20) is an immediate precursor of C30 and C40 carotenoids, and GGPP synthase (GGPS) catalyzes the formation of GGPP. Phytoene is the first carotenoid produced, from the condensation of two molecules of GGPP, a process catalyzed through the bifunctional enzyme phytoene-lycopene synthase [9]. Next, phytoene desaturase performs four successive desaturations to generate lycopene. The cyclization of lycopene through phytoene-lycopene synthase produces β-carotene [10]. Subsequently, β-carotene is oxidized at both ends through the cytochrome P450 enzyme, astaxanthin synthase [11]. This reaction requires the accessory activity of cytochrome P450 reductase (CPR) as an electron donor [12]. However, although astaxanthin biosynthesis has been elucidated at genetic level, the complex regulatory mechanisms controlling this process remain unknown.

In *X. dendrorhous*, it has been proposed that astaxanthin fulfills a cellular defense role against oxidative damage; indeed, the pigment production is induced through the generation of reactive oxygen species (ROS) [13]. Moreover, among others factors, the carbon source and oxygen level can influence the amount of pigment synthesized [14]. Indeed, astaxanthin production increases under aerobic metabolism and decreases during fermentative growth [15]. In *X. dendrorhous*, carotenogenesis is growth-associated and depends on the growth conditions, such as the carbon source [16-18]. Notably, carotenogenesis is induced at the beginning of the stationary growth phase when the yeast is cultured in glucose-supplemented medium. In contrast, when succinate is used as the carbon source, the carotenogenesis is induced early during the exponential growth phase [18]. Additionally, *X. dendrorhous* carotenoid production is increased when cultured using a non-fermentable carbon source, such as succinate or ethanol [16,18].

These observations might reflect the transcriptional control of carotenogenesis in glucose medium, but this mechanism is not applicable for growth in succinate medium because there is not a direct association between carotenogenesis induction and the maximum expression levels of carotenogenic genes [18]. This behavior suggests the existence of additional carotenogenic regulatory mechanisms associated with growth, likely at the enzymatic level.

In the present study we integrated metabolic profiling and the protein abundance data to examine the influence of fermentable and non-fermentable carbon sources (glucose and succinate, respectively) on yeast and we identified the metabolites and proteins associated with the carotenogenesis. To this end, two-dimensional gel electrophoresis (2-DE) coupled with matrix-assisted-laser-desorption/ionization time-of-flight mass spectrometry (MALDI-TOF MS) and gas chromatography–mass spectrometry (GC-MS), were employed for protein and metabolite determination, respectively.

## Results

### Growth and carotenoid production in glucose- and succinate- supplemented media

**Figure 1:**
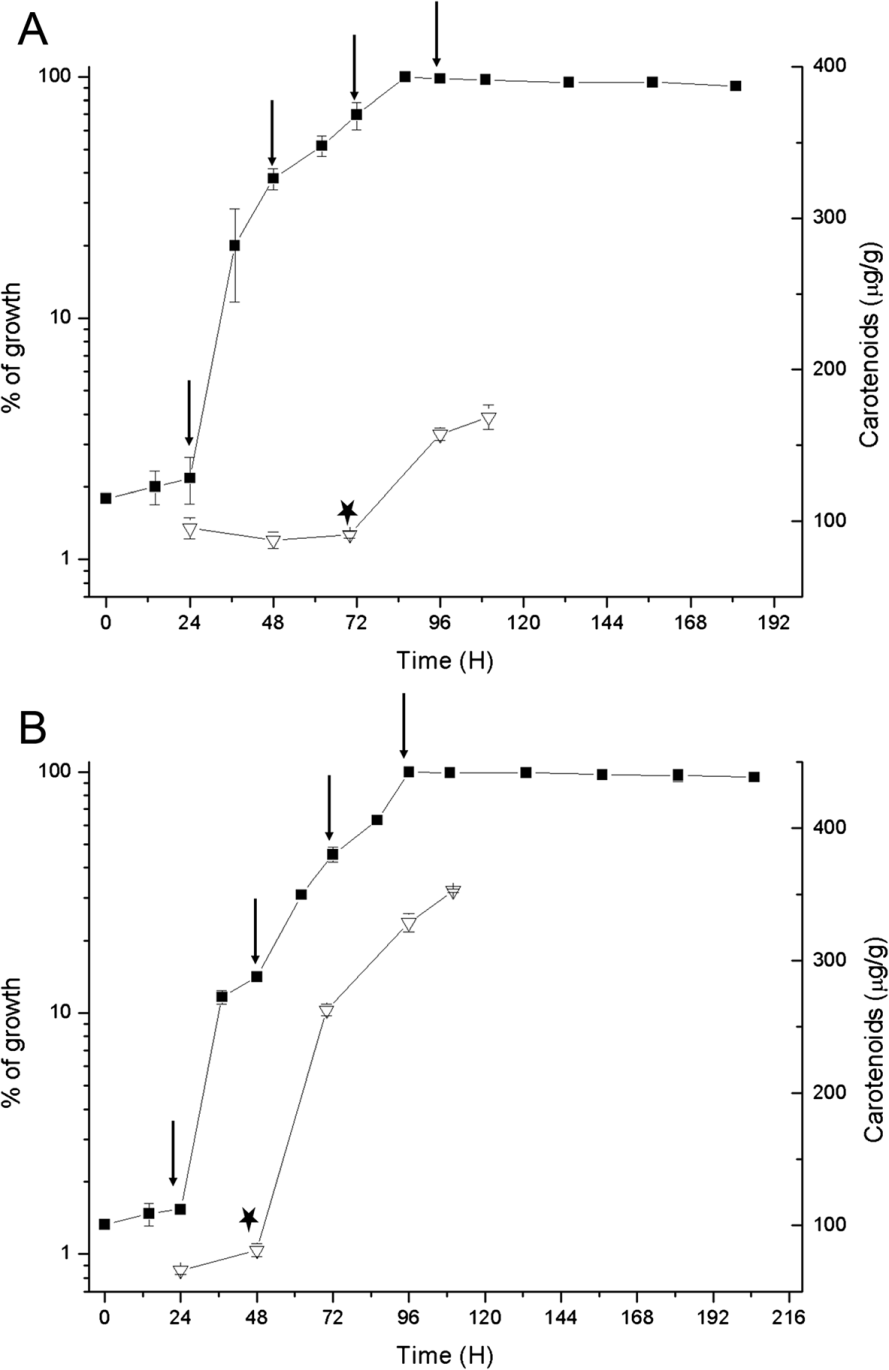
Growth and carotenoid production during the growth cycle in *Xanthophyllomyces dendrorhous*. The UCD 67–385 strain was grown in MM supplemented with 2% of glucose **(A)** or succinate **(B)**. (■) Represents the percentage of growth. The maximum absorbance (560 nm) value was considered as 100% of growth and the value for each time point, was calculated as a percentage of maximum growth. The means ± SD of the values obtained from three independent cultures are shown. The vertical arrows indicate the harvest times for the assays (24, 48, 70 and 96 h, which corresponded to lag, early exponential, late exponential and stationary phases of growth, respectively). (∇) Represents the total carotenoids. (_*_) Indicates the induction of carotenoid biosynthesis.

In a previous study, we determined that carotenogenesis in *X. dendrorhous* is favored when the yeast is cultured in succinate than in other non-fermentable carbon sources, such as xylose or sodium acetate [18]. To compare the effect of fermentable and non-fermentable carbon sources, the *X. dendrorhous* UCD 67–385 strain was cultured in minimal medium (MM) supplemented with 2% of glucose or succinate. To obtain cellular extracts for all experiments, the cells were harvested from cultures at lag, early exponential, late exponential and stationary growth phases (Figure 1).

Consistent with the previous study [18], the carotenoids content when the yeast was cultured in succinate was 350 μg/g (dry yeast weight), which is more than twice the value obtained when cultured in glucose-supplemented medium (167 μg/g) (Figure 1). Using reverse phase high-pressure liquid chromatography (RP-HPLC), we determined that astaxanthin is the major carotenoid produced under both culture conditions, with no significant differences in composition (data not shown). Thus, in succinate medium, *X. dendrorhous* astaxanthin production is enhanced.

Additionally, in glucose-supplemented medium, carotenogenesis was induced at the beginning of the stationary growth phase, whereas in succinate, this process was induced earlier during the exponential growth phase (Figure 1) and steadily increased until the stationary phase. This result indicates that carotenogenesis is associated with growth in succinate [18].

### *X. dendrorhous* proteome in fermentable and non-fermentable carbon sources

For the proteomics analyses, triplicate protein extracts from yeast grown at each growth phase (extracted from independent cultures) were subjected to 2-DE, and the protein profiles were analyzed using PDQuest software (version 7.1.1, Bio-Rad). Student’s t-test (p < 0.02) was used to identify significant changes in protein abundance. To control for the false discovery rate (FDR), defined as the expected proportion of false positives among the proteins with significant abundance changes, we applied the Benjamini-Hochberg multiple testing correction (p < 0.05).

In general, the protein profile did not show major differences between either carbon sources at different stages of growth (see Additional file 1: Figure S1). Approximately 1,200 protein spots were extracted from the gels and identified using MALDI-TOF MS. In total, 886 spots were identified by peptide mass fingerprint (PMF), corresponding to 329 non-redundant proteins (see Additional file 2: Table S1). Notably, 24 proteins were identified in multiple spots, likely representing protein isoforms due to post-translational modifications.

The identified proteins were classified according to biological functions (Additional file 2: Table S1). Approximately 60% of the proteins were associated with central metabolism including amino acids, carbohydrates and redox proteins, which were the most abundant.

Student’s t-tests with Benjamini-Hochberg multiple testing correction (p < 0.05) were applied to identify significant differences in the intensity of the proteins spots. First, the protein abundance in the early exponential, late exponential and stationary phases of growth relative to the lag phase was evaluated in cultures under each carbon source. In glucose medium, 37 proteins with differential abundance were identified (Additional file 3: Table S2A). Notably, several proteins showed high abundance during the late exponential phase, corresponding to carotenogenesis induction in glucose medium. For example, the phosphoglucomutase protein (spot 7519) exhibited a 3-fold higher intensity during the late exponential phase relative to the intensity observed during the lag phase. Among the redox proteins, the monooxygenase (spot 5703), Mn-superoxide dismutase (MnSOD) (spot 7108) and cytochrome P450 protein (spot 5315), showed similar patterns (p < 0.02). In addition, the carotenogenic enzymes diphosphomevalonate decarboxylase (spot 6308) and phytoene squalene synthase (spot 4515) also showed similar patterns. However, the abundance profile of some carotenogenic enzymes, like mevalonate kinase (MK), phosphomevalonate kinase and diphosphomevalonate decarboxylase, did not correspond to any sequential enzymatic function within the carotenogenic pathway. These last three enzymes are not exclusively carotenogenic as they belong to the MVA pathway, which also provides metabolite precursors to other pathways [7].

In contrast, 46 proteins exhibited differential abundance during the analyzed phases of growth when cultured in succinate (Additional file 3: Table S2B). Unlike the patterns observed in glucose-supplemented cultures, a high abundance of several proteins involved in carbohydrate metabolism were observed during carotenogenesis induction in succinate corresponding to the early exponential growth phase. For example, some tricarboxylic acid cycle (TCA) and glycolysis enzymes such as the citrate synthase (spot 6503), succinyl-CoA synthase (spot 5509), succinate dehydrogenase (spot 6610), glyceraldehyde 3-phosphate dehydrogenase (GAPDH) (spots 4312 and 5314) and phosphoglucomutase (spot 7519). A total of 3 redox proteins, monooxygenase (spot 5320), cytochrome P450 (spot 5315) and thioredoxin (spot 9127) were highly abundant during carotenogenesis induction. Among carotenogenic proteins, astaxanthin synthase (spot 7501), MK (spot 4609), and GGPS (spot 4303) were highly abundant during the early exponential phase. Similar to the patterns observed in glucose cultures, these carotenogenic proteins did not show a sequential abundance profile.

When the proteins patterns obtained from both carbon sources were compared at the same growth stage, 70 proteins showed significant abundance differences (Table 1). In the carbohydrate group, most of the enzymes associated with glycolysis (glucokinase (spot 6609), phosphoglycerate kinase (spot 4201) and pyruvate dehydrogenase (spot 6403)) and the pentose phosphate (PP) pathway (glucose-6-phosphate dehydrogenase (spot 5617) and transaldolase (spot 2213)) were more abundant in glucose. However, the enzymes associated with the TCA cycle (citrate synthase (spot 6503), succinate dehydrogenase (spot 6610), succinyl-CoA synthase (spot 5509) and malate dehydrogenase (spot 7206)) and lipid metabolism (fatty acid synthase (spot 6604) and acyl-CoA synthase (spot 4603)) showed higher abundance in succinate. For the redox group, the increased abundance of monooxygenase (spots 4713 and 5703), dehydrogenase SDR (spot 5202), glutathione-disulfide reductase (spot 7618), and oxidoreductase (spot 4310) were observed in succinate cultures. In the same group, thioredoxin peroxidase 2 (TSA2) (spot 7105), alcohol dehydrogenase (ADH) (spot 5208) and MnSOD (spot 7108) proteins were more abundant in glucose medium. With respect to carotenogenesis, a significantly high abundance of MVA pathway (MK (spot 4609) and phosphomevalonate kinase (spot 3517)) and late carotenogenesis (astaxanthin synthase (spot 7501) and CPR (spot 7311)) enzymes was observed in succinate cultures.

**Table 1:** **Relative changes in the yeast protein abundance when cultured in succinate versus glucose**

		^**c**^ **Fold-change succinate/glucose**			
^**a**^ **SSP**	^**b**^ **Assignment**	**L**	**EE**	**EL**	**S**
	**Cellular Processes: Transport and Motor Proteins**				
6813	Putative coatomer subunit alpha	2.22	3.53	2.99	**2.32**
8703	Myosin -associated protein	**26.33**	**2.34**	3.83	2.58
8711	Myosin -associated protein	**16.31**	2.01	3.47	2.13
7403	KIP1 kinesin- related protein	**4.68**	−1.20	1.01	1.42
2503	Alpha-tubulin	*3.92*	−3.12	2.70	2.38
	**Environmental Information Processing and Signal Transduction**				
5515	Negative regulator of the PHO system. Ser-thr kinase	**−5.15**	−1.20	−2.03	−1.04
3808	Serine/threonine-protein phosphatase PP1-1	1.09	*5.86*	1.48	1.49
9206	Ribosomal_L15	**−9.72**	−10.86	−6.31	−30.40
8104	Protein PXR1	*−18.08*	−21.03	−22.68	−24.75
5417	Eukaryotic translation initiation factor 3 subunit H	*−18.02*	−1.63	2.41	−3.29
4411	Pre-mRNA-splicing factor	**−49.64**	−5.13	−3.64	1.09
7815	Mediator of RNA polymerase II transcription sub.14	3.62	3.10	2.24	*2.05*
5602	Hypothetical protein. ATP-binding, Chaperone	*−5.05*	1.17	−2.18	−1.25
2603	HSP60	**5.25**	−1.50	−1.09	−1.01
6411	Transcription factor RfeF, putative	**22.00**	3.95	2.84	3.20
7109	GTP-binding nuclear protein RAN	*−2.19*	−1.69	−1.26	−1.07
	**Metabolism**				
3715	HSP 70	1.04	*2.37*	1.00	1.18
7812	Vacuolar membrane ATPase subunit a precursor	**2.66**	2.61	4.24	1.83
8504	Glutathione S-transferase Gst3	**5.43**	−1.65	1.88	3.71
	**Metabolism: Lipid and Carbohydrate**				
2523	CQ798506 NID. Acetyl-CoA carboxylase, cytosolic	*−4.12*	−1.57	−2.23	−2.88
4603	ADR052 Wp. Simil acyl-CoA synthetase	**3.96**	3.75	3.98	7.02
6604	Fatty acid synthase	**14.69**	2.32	*19.30*	2.64
2319	Acetyl-CoA synthetase	*−6.54*	−2.51	−19.85	−1.18
6503	Citrate synthase	1.07	*6.07*	*3.12*	2.24
6609	Glucokinase	**−5.48**	−3.12	−4.63	**−8.47**
4201	Phosphoglycerate kinase	*3.02*	−2.30	−1.04	1.74
5314	GAPDH	**11.61**	−1.46	1.51	−2.33
2213	Transaldolase	1.03	−2.83	*−2.42*	−2.21
6610	Succinate dehydrogenase (ubiquinone)	−4.25	1.55	*3.22*	*1.74*
6403	Pyruvate dehydrogenase	**−27.44**	−1.20	2.54	−1.55
5509	Succinyl-CoA synthetase beta subunit	−1.15	**16.26**	*17.02*	6.02
3206	NADP+ malate dehydrogenase	**36.04**	1.90	*38.06*	3.81
4605	Pyruvate decarboxylase	**5.43**	3.29	3.21	*2.90*
4519	Glucose-6-phosphate isomerase	−5.39	*−26.97*	−1.06	3.10
0604	6-phosphogluconate dehydrogenase	**6.28**	−1.42	1.76	2.69
3331	Enolase	1.07	*−3.59*	−1.64	−1.75
4717	Aldehyde dehydrogenase [NAD(P)+]	*4.27*	2.02	−1.93	6.80
7110	Ribose-5-phosphate isomerase	1.42	−2.02	*9.29*	−2.32
5617	Glucose-6-P dehydrogenase	**−5.94**	−5.64	−10.98	−2.08
7206	Malate dehydrogenase	**7.82**	1.70	3.24	4.74
6516	Acyl-CoA carboxylate CoA-transferase	6.07	**7.73**	8.02	4.43
6109	Endo-1,3(4)-beta-glucanase -glucanase	*−4.62*	−1.23	−2.28	−2.36
	**Metabolism: Secondary metabolite/carotenoid biosynthesis**				
4609	Mevalonate kinase	13.23	3.51	**71.38**	10.07
5717	Squalene synthase	**−2.19**	1.28	1.01	−1.26
5303	Prenyltransferase	*1.46*	−1.17	**−5.57**	−1.11
6308	Diphosphomevalonate decarboxylase	*1.62*	1.25	−1.27	−1.30
3517	Phosphomevalonate kinase	1.14	**4.42**	1.99	2.43
7501	Astaxanthin synthase	−1.15	**1.50**	3.62	2.30
7311	Cytochrome P450 reductase (crtR) gene	*1.62*	1.40	−1.11	−2.87
	**Metabolism: Redox**				
4713	Monooxygenase	*1.68*	2.28	2.20	1.78
5703	Monooxygenase	**2.18**	3.91	1.45	2.05
5208	Alcohol dehydrogenase	−3.91	−2.74	*−5.20*	−8.99
5202	Dehydrogenases with different specificities SDR	**3.53**	1.62	1.67	1.24
7108	Mn superoxide dismutase	**−27.58**	−6.57	−40.12	−1.10
7105	Peroxiredoxin TSA2.	*−41.82*	−18.13	**−57.80**	−2.93
4310	Oxidoreductase	1.10	*3.09*	3.60	1.57
5320	Monooxygenase, putative	**19.98**	1.81	26.11	22.52
7618	Glutathione-disulfide reductase	**33.21**	3.49	1.86	1.42
2210	Short-chain dehydrogenase/reductase SDR	*−10.14*	−2.70	21.55	1.26
5313	Probable thioredoxin peroxidase	*−8.84*	1.07	−4.86	*−4.04*
	**Metabolism: Amino acid**				
7811	Mitochondrial isoleucyl-tRNA synthetase	**1.94**	2.25	−1.13	1.66
7210	RIB40 genomic DNA. Methionyl-tRNA formyltransferase	**10.14**	−1.18	1.78	2.03
7816	Kynurenine 3-monooxygenase	6.59	4.93	**2.74**	2.80
7307	Aspartate aminotransferase putative	*4.58*	1.60	1.03	−2.50
2628	Adenosylhomocysteinase	**−27.35**	1.36	−3.14	−2.53
3515	Delta-1-pyrroline-5-carboxylate dehydrogenase	−2.66	−2.32	*5.77*	1.93
	**Unknown**				
7715	UPF0553 protein C589.05c.	**5.54**	4.35	1.38	**−1.51**
5304	Conserved hypothetical protein	**−6.74**	−1.43	−2.32	−2.82
3615	YALI0F00616p	**−5.60**	1.39	32.23	2.13
0310	Predicted protein	*3.56*	**−4.53**	−4.39	−1.57
3002	Hypothetical protein	**−2.89**	−2.51	3.24	2.19
6111	Hypothetical protein	*−14.03*	1.15	4.27	−1.05

^a^SSP numbers were assigned using the PDQuest software analysis. ^b^Identifications were obtained using the Swiss-Prot and KEGG Pathways databases and contigs of *X. dendrorhous* genomic DNA. ^c^Mean fold changes in succinate compared with glucose. Statistical significance was estimated by *t*-test (p <0.02) that is shown in italics and the Benjamini-Hochberg (p <0.05) correction shown as bold values. Abbreviations: L: lag phase, EE: Early exponential, EL: Late exponential, S: Stationary.

### Metabolite profiling of *X. dendrorhous* cultured in fermentable and non-fermentable carbon sources

To compare the relative amounts of metabolites in the different samples collected, the response ratio of each metabolite normalized to the internal standard ribitol was transformed to log_2_. Significant differences (t-student test corrected by Benjamini-Hochberg multiple testing correction p < 0.05) between samples from succinate and glucose cultures were evaluated and the complete datasets are presented in Additional file 4: Table S3.

**Figure 2:**
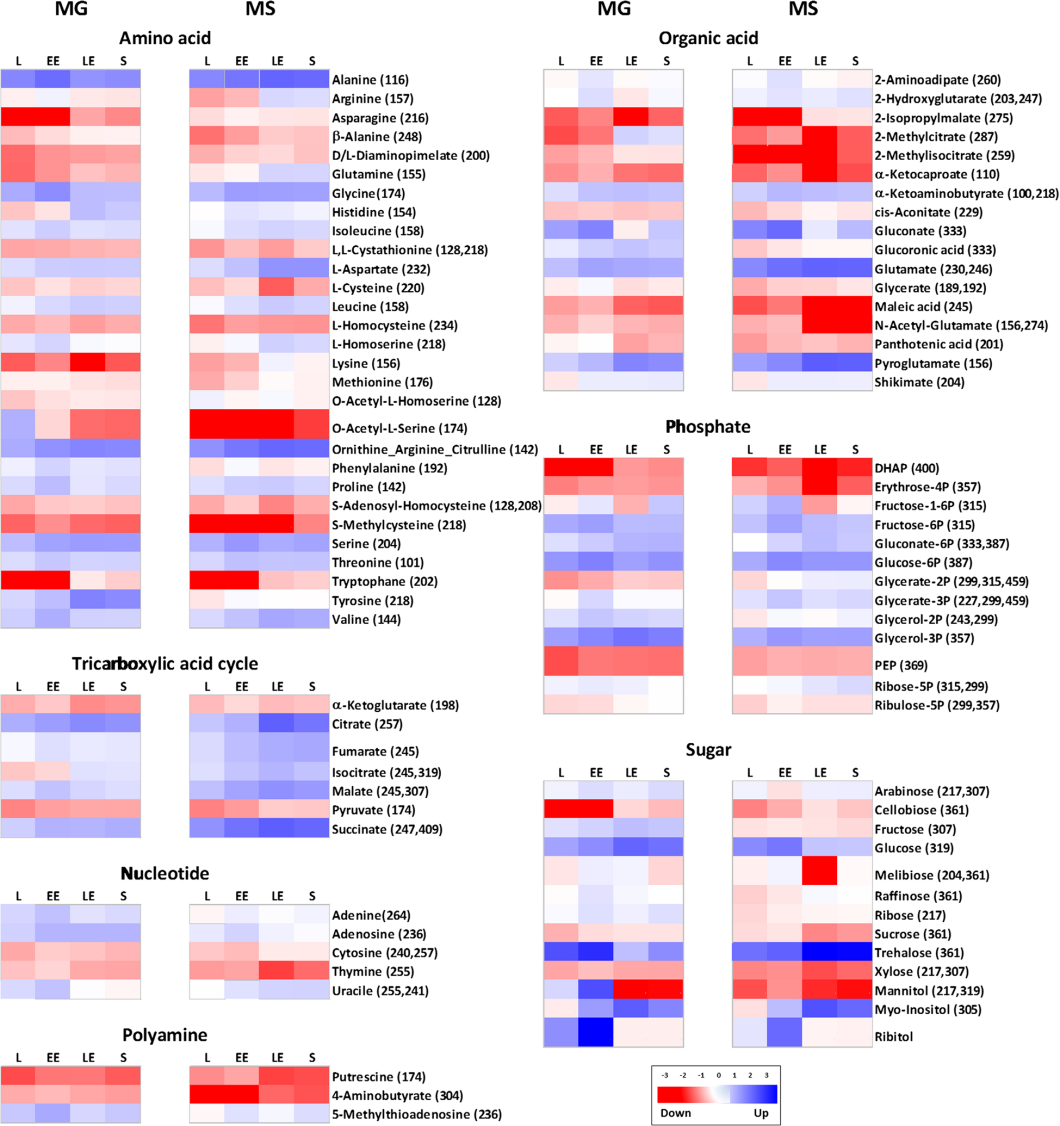
Metabolite profile of *X. dendrorhous*. For each metabolite in glucose (MG) or succinate (MS), the response ratio was normalized to Log_2_. The m/z values of selected ions used for quantification are shown in parentheses. Each column graphic represents the growth phases studied. L: lag, EE: early exponential, LE: late exponential, S: stationary. Red or blue color indicates that the metabolite content is decreased or increased, respectively. Metabolites were grouped according to the KEGG database. Abbreviations: TCA: Tricarboxylic acid cycle, DHAP: dihydroxyacetone phosphate, PEP: phosphoenolpyruvate.

A total of 92 polar metabolites associated with primary metabolism were analyzed, and the functional classification is shown in the heat map in the Figure 2. The metabolomes of different yeast samples cultured in glucose or succinate as carbon sources were compared at the same growth stage, and the highest differences were observed in TCA cycle intermediates, sugars and some amino acid metabolic pools.

Particularly, trehalose had the highest signal intensity observed under both growth conditions, although this sugar was significantly more abundant in succinate.

Over the entire course of cultivation in succinate, significantly high abundance of the metabolic pools of TCA cycle intermediates and amino acids derivate were observed (Figure 2). Similarly, the metabolites of gluconeogenesis and the glyoxylate cycle were more abundant in succinate.

In glucose cultures, the metabolites and amino acids derived from the initial steps of glycolysis and PP pathway were more abundant than those observed in succinate.

### Metabolome and proteome mapping

**Figure 3:**
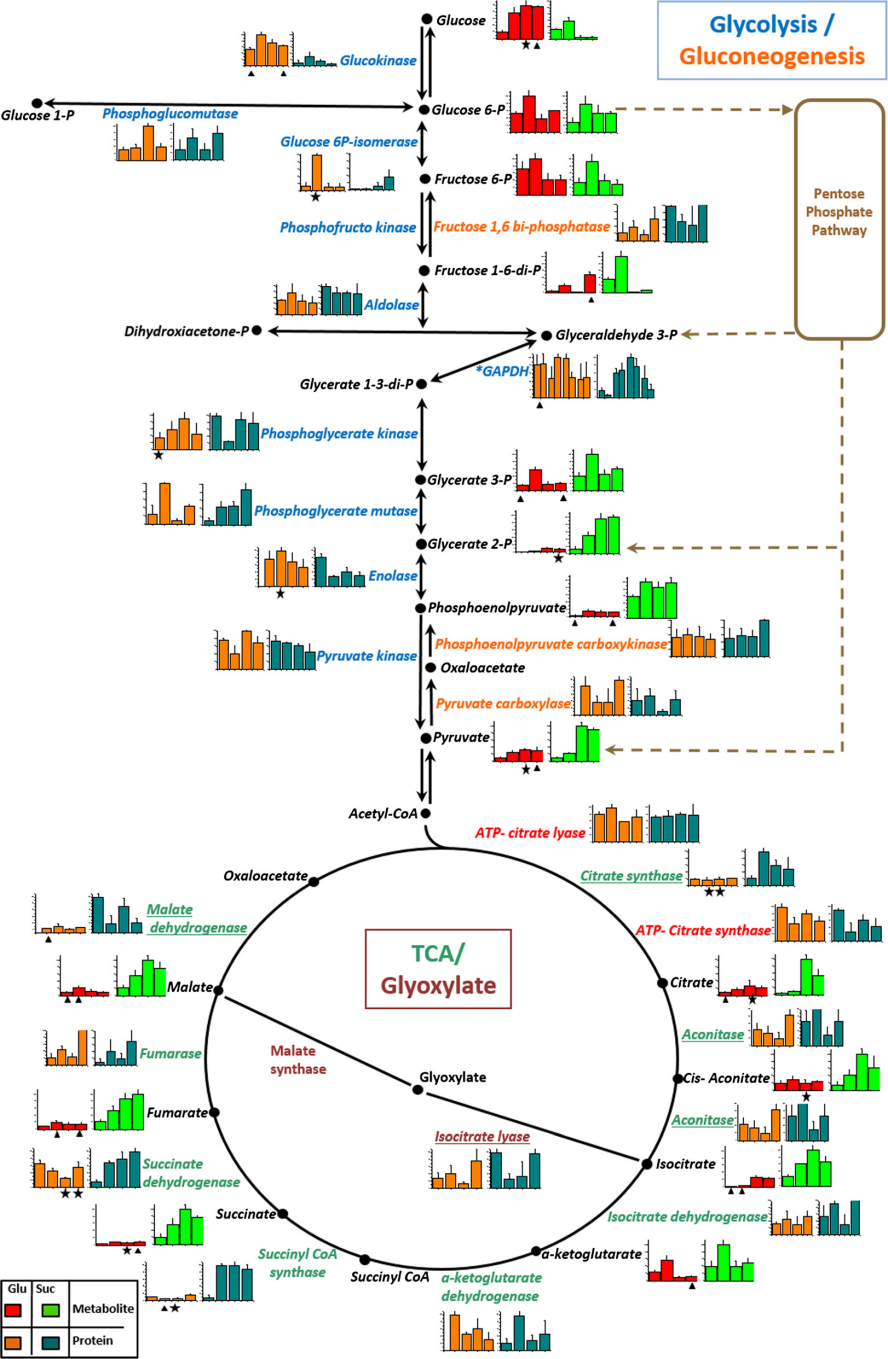
Protein and metabolite levels in the glycolysis, gluconeogenesis, glyoxylate and TCA pathways. According to the carbon source the mean value and SD of abundance for each protein, and the response ratio for each metabolite were normalized to a value of 100. Each column graphic represents the growth phases studied (from left to the right: lag, early exponential, late exponential and stationary). In the column charts a color code was included to differentiate proteins and metabolites found when *X. dendrorhous* was cultured with the different carbon sources: proteins in glucose (orange), proteins in succinate (cyan), metabolites in glucose (red) and metabolites in succinate (green). The pathways were adapted from the KEGG database. In the metabolic pathways, names are written in a color letters: metabolites (black), glycolysis proteins (blue), gluconeogenesis proteins (orange), glyoxylate proteins (gray) and TCA proteins (green). Proteins involved in both, the glyoxylate and TCA cycle, are underlined and cytosolic proteins related to acetyl-CoA formation are indicated in red. An asterisk beside the protein name and in the multiple column charts indicates that the protein probably suffers post-translational modifications as it was identified in multiple spots. Statistical significant differences between samples from different carbon sources at the same growth phase are represented as stars (*t*-test p < 0.02) and triangles (Benjamini-Hochberg correction p < 0.05). Abbreviations: P: phosphate, TCA: tricarboxylic acid cycle.

**Figure 4:**
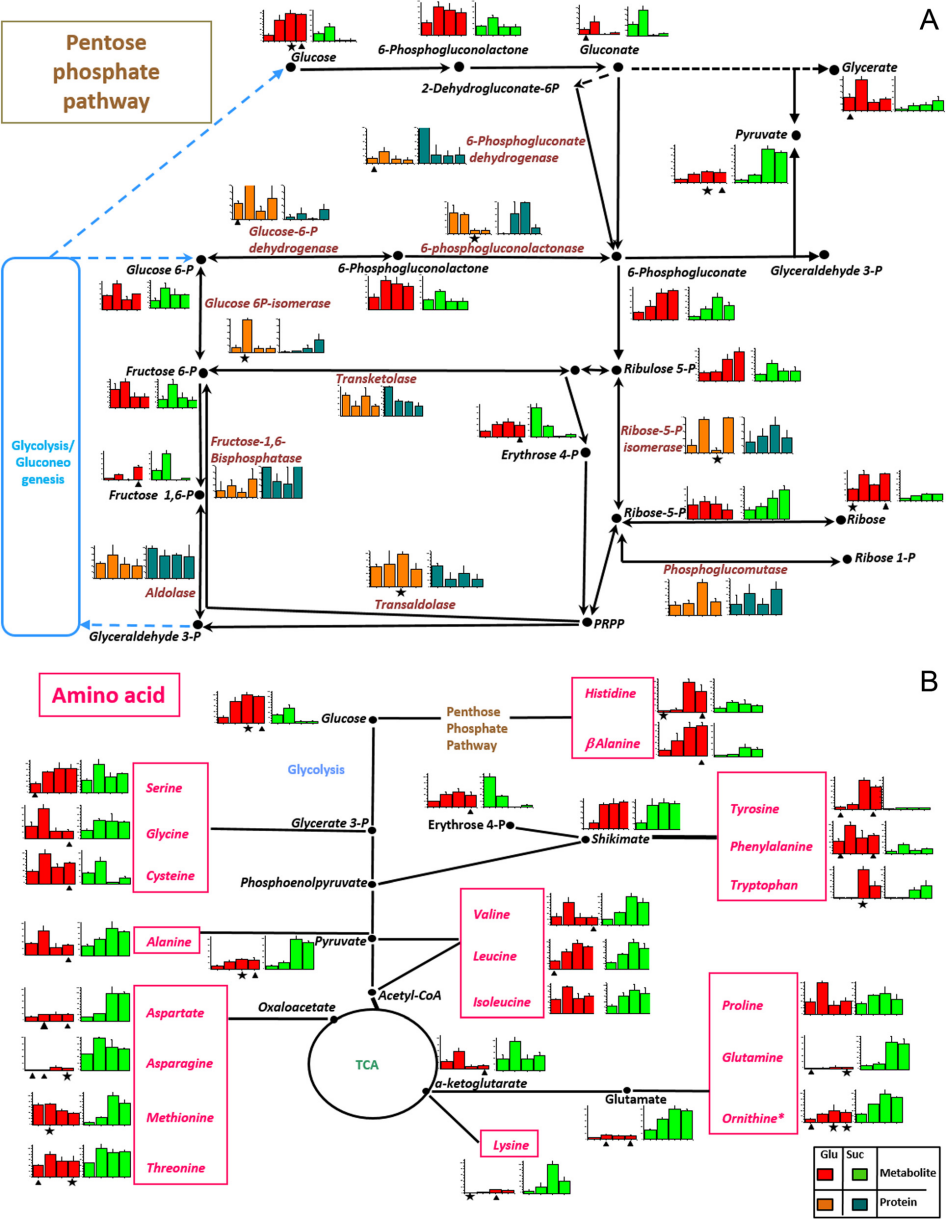
Protein and metabolite level in the pentose phosphate and amino acid pathways. **A**: Pentose phosphate and **B**: amino acid pathways. According to the carbon source, the mean value and SD of abundance for each protein and the response ratio for each metabolite were normalized to a value of 100. Each column graphic represents the growth phases studied (from left to the right: lag, early exponential, late exponential and stationary). In the column charts a color code was included to differentiate proteins and metabolites found when *X. dendrorhous* was cultured with the different carbon sources: proteins in glucose (orange), proteins in succinate (cyan), metabolites in glucose (red) and metabolites in succinate (green). The pathways were adapted from the KEGG database. In the metabolic pathways, names are written in a color letters: metabolites (black), PP pathway proteins (coffee) and amino acids (pink). Statistical significant differences between samples from different carbon sources at the same growth phase are represented as stars (*t*-test p < 0.02) and triangles (Benjamini-Hochberg correction p < 0.05). Abbreviations: PRPP: 5-phosphoribosil diphosphate, P: phosphate, TCA: tricarboxylic acid cycle.

**Figure 5:**
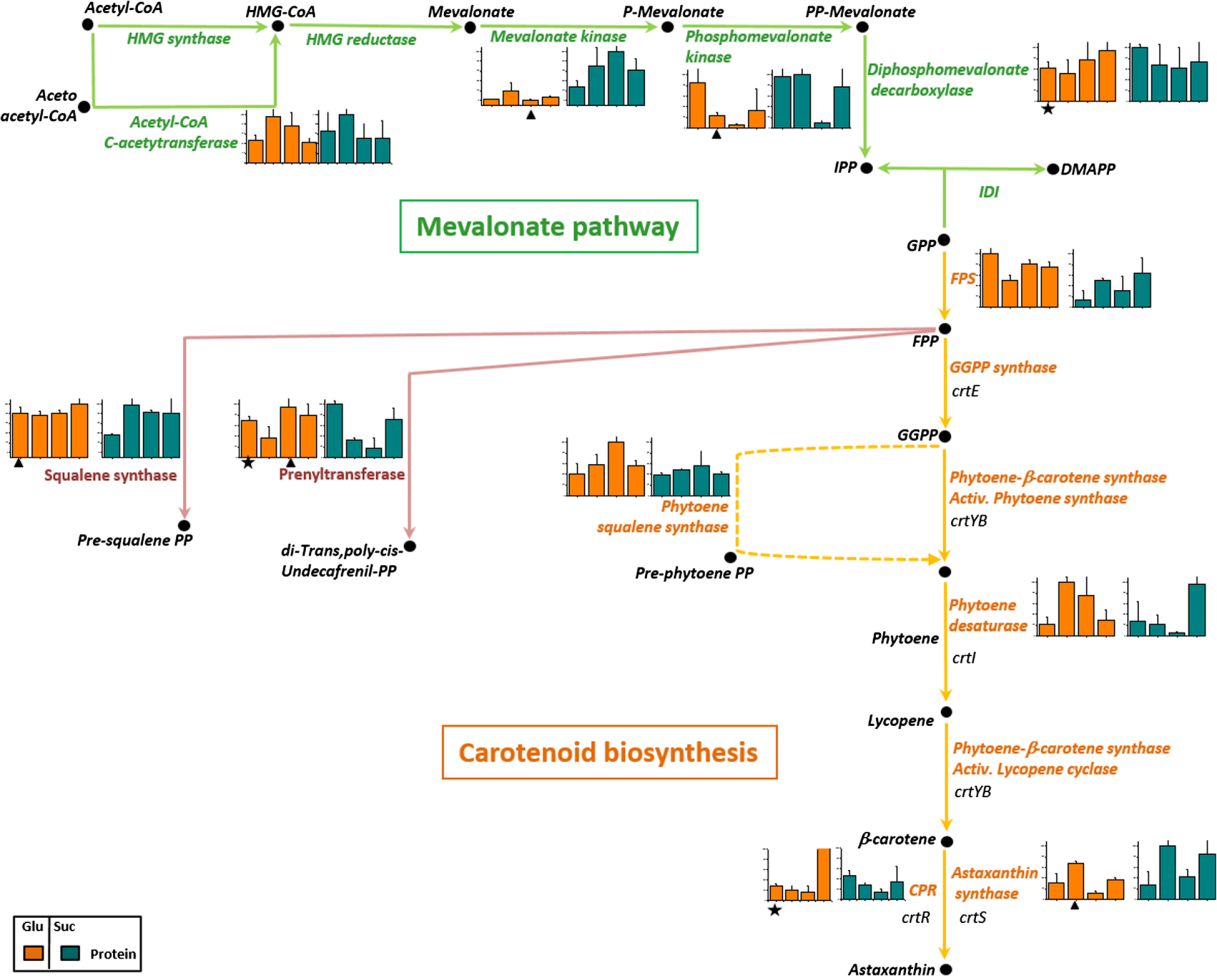
Protein levels in mevalonate pathway and carotenogenesis. According to the carbon source the mean value and SD of abundance for each protein was normalized to a value of 100. Each column graphic represents the growth phases studied (from left to the right: lag, early exponential, late exponential and stationary). In the column charts a color code was included to differentiate proteins found when *X. dendrorhous* was cultured with the different carbon sources: proteins in glucose (orange) and proteins in succinate (cyan). The pathways were adapted from the KEGG database. In the metabolic pathways, names are written in a color letters: mevalonate proteins (green), carotenogenesis proteins (orange), related pathway proteins (purple), carotenoids and intermediates (black). The carotenogenic genes are illustrated in italic letters. Statistical significant differences between samples from different carbon sources at the same growth phase are represented as stars (*t*-test p < 0.02) and triangles (Benjamini-Hochberg correction p < 0.05). Abbreviations: HMG: hydroxymetylgluraryl, CoA: coenzyme A, P: phosphate, IPP: isopentenyl pyrophosphate, IDI: isopentenyl diphosphate isomerase, DMAPP: dimethylallyl pyrophosphate, GPP: geranyl pyrophosphate, FPP: farnesyl pyrophosphate, GGPP: geranylgeranyl pyrophosphate, CPR: cytochrome P450 reductase.

To correlate the identified proteins and metabolite dynamics, we adapted the principal carbon, amino acid and carotenogenesis pathways from the KEGG database (Figures 3, 4 and 5, Additional file 5: Figure S2 and Additional file 6: Figure S3). To facilitate comparison, the mean values of the metabolites or proteins, represented as the response ratio transformed to log2 or the normalized-intensity value, respectively, were normalized to 100 according to the growth phase. The normalized data for glucose or succinate were shown in a column diagram including the standard deviation and Student’s t-test adjusted results by Benjamini-Hochberg multiple testing correction. For the proteins, the multiple column charts represent the presence of post-translational modification (proteins that were represented by multiple spots) on pathways figures.

The Additional file 5: Figure S2A, shows the sugar metabolism pathway. In glucose, the clear high abundance of α-glucosidase and glucokinase enzymes and the metabolites D-glucose, D-fructose and D-mannitol are evidence of the major availability of this carbon source.

The other identified enzymes did not show significant differences during growth in both carbon sources. Notably, a majority of the identified metabolites were more abundant in glucose cultures, likely reflecting the post-translational mechanisms regulating enzymatic activities. In succinate cultures, the increased abundance of the enzyme trehalase was observed during the stationary phase, associated with a significant increase in the sugar trehalose. Interestingly, the accumulation of this sugar has been associated with the oxidative stress response [19].

In general, glycolysis and gluconeogenesis pathway enzymes did not show significant differences in abundance (Figure 3). However, a clear metabolite tendency was observed: in glucose cultures, early glycolysis metabolites were increased, while metabolites generated during the final glycolysis reactions were significantly higher in succinate cultures (Figure 3).

For the PP pathway, a direct correlation between the abundance of the proteins involved and the generated metabolites was not observed. For example, some enzymes (transaldolase and glucose 6-P dehydrogenase) were markedly increased in glucose. However, 6-phosphogluconate dehydrogenase and ribose 5-phosphate isomerase were significantly abundant in succinate cultures (Figure 4A). In general, all metabolites were more abundant in glucose and consistent with previous studies [20,21], the regulation of the abundance of some proteins in this pathway such as glucose 6-P dehydrogenase was observed.

Regarding pyruvate metabolism (Additional file 6: Figure S3A), pyruvate dehydrogenase, which facilitates the formation of acetyl-CoA in the mitochondria, was more abundant at the beginning of the growth curve when cultured in glucose, but during the late exponential growth phase, a notable increase in succinate was observed. This behavior is likely associated with increased acetyl-CoA generation through glycolysis in glucose cultures, and pyruvate production through gluconeogenesis in succinate cultures. Other pyruvate metabolism enzymes such as acetyl-CoA synthase and ADH, were more abundant when *X. dendrorhous* was cultured in glucose (Additional file 6: Figure S3A), likely reflecting the influence of the carbon source and its role in fermentation reactions.

Because succinate is directly incorporated into the TCA cycle, the enzymes and metabolites from the TCA and glyoxylate cycles were significantly more abundant in succinate cultures, suggesting that the carbon source affects the high level of metabolite production in this pathway under aerobic growth conditions (Figure 3). Correspondingly, the high abundance of some oxidative phosphorylation enzymes such as ATP synthase α, NADH dehydrogenase and metabolites such as succinate and fumarate, were detected in succinate cultures (Additional file 6: Figure S3B). Consistently, some enzymes involved in metabolism of the fatty acids and glycerophospholipids such as fatty acid synthase (FAS) and acyl-CoA synthase, showed significant abundance in succinate cultures (Additional file 5: Figure S2B).

Figure 4B shows the comparison between the abundance of amino acids, enzymes and metabolites that precedes the synthesis of these molecules in the related pathways. For instance, the amino acids generated from the glycolysis and PP pathways such as β-alanine, cysteine and tryptophan were more abundant in glucose cultures. However, the amino acids derived from the TCA cycle, such as lysine, aspartate and glutamine, showed higher abundance in succinate cultures.

In *X. dendrorhous*, carotenoid synthesis begins with the formation of IPP from acetyl-CoA through MVA. From this pathway, MK showed a remarkable increase in succinate cultures. Another two enzymes involved in sequential steps in this pathway, phosphomevalonate kinase and diphosphomevalonate decarboxylase, also showed significant increments in succinate cultures during the early exponential and lag phases of growth, respectively (Figure 5).

The enzymes involved in the production of carotenogenesis precursors such as prenyltransferase and squalene synthase, which is involved in ergosterol synthesis, were more abundant in glucose cultures (Figure 5). Moreover, the enzymes involved in the last steps of carotenogenesis in *X. dendrorhous*, astaxanthin synthase and CPR, were significantly higher in succinate cultures (Figure 5) suggesting that the increased production of astaxanthin in yeast grown in succinate might depend on the availability of the substrates (acetyl-CoA) and the abundance of these two enzymes.

## Discussion

### Carbon source influence on carotenogenesis and central metabolism in *X. dendrorhous*

In the present study, we provided a detailed analysis of the influence of carbon sources (i.e., glucose and succinate) in the central metabolism of the yeast *X. dendrorhous*, revealing noticeable differences between the studied metabolic pathways, including glycolysis, PP pathway, TCA and glyoxylate cycles and lipid metabolism.

Overall, a direct relation between the carbon source and the identified metabolites was observed. For instance in glucose cultures, the metabolites involved in the initial steps of glycolysis and in the PP pathway were more abundant (Figures 3 and 4A), suggesting enzymatic regulation through fructose 1–6 diphosphate [22].

In the present study, regulation via the abundance of glycolytic enzymes including glucokinase, GAPDH and enolase, was observed (Figure 3). Consistently, when *S. cerevisiae* is cultured in glucose or a non-fermentable carbon source the same trend has been described [23-25]. Additionally in our study the enzyme GAPDH was identified in multiple spots under both carbon sources conditions, but the spots did not show a similar abundance pattern (Figure 3), suggesting a post-translational regulation mechanism such as phosphorylation. Other carbohydrate metabolism enzymes regulated through phosphorylation during yeast growth with different carbon sources and under stress conditions have been identified [19,23-26].

The PP pathway is regulated through competitive inhibition [27] and post-translational modifications via phosphorylation [20,21]. In addition this pathway is considered as the primary source of NADPH, which is markedly important in the oxidative stress response to remove ROS [28]. Thus, two PP pathway enzymes, transketolase and transaldolase, were overexpressed in *S. cerevisiae* to overcome the SOD deficiency [29]. Our results show that phosphoglucomutase (spot 7519), which is involved in the PP pathway, glycolysis and trehalose synthesis, was upregulated during carotenogenesis induction in cultures with both carbon sources (Additional file 3: Table S2A and B). Interestingly, an increase in this enzyme in response to H_2_O_2_ treatment has also been observed [19]. Considering that *X. dendrorhous* requires oxygen and NADPH for carotenogenesis, our results suggest that the high activity of this metabolic pathway might be required to remove ROS in the absence or with low levels of another enzymatic defense mechanism.

It has been previously reported that the addition of succinate increases the emission of H_2_O_2_ in mitochondria leading to a higher respiration rate [30]. In the present study, a direct effect on yeast growth in succinate medium with increase in the metabolites and enzymes of the TCA and glyoxylate cycles (Figure 3), oxidative phosphorylation (Additional file 6: Figure S3B) and amino acid derivatives (Figure 4B), was observed. Furthermore, an increased production of carotenoids was obtained reflecting the direct incorporation of succinate into the TCA cycle, generating more substrates that could increase the respiratory rate in the cell leading to ROS accumulation and thereby, favoring carotenoid production.

Regarding lipid metabolism, *X. dendrorhous* is an oleaginous yeast species [31] with a high flux of PP pathway and high enzymatic activity of the citrate lyase and phosphoketolase [32]. Furthermore, citrate accumulation has been associated with lipid accumulation reflecting the low or absent activity of the isocitrate dehydrogenase in mitochondria. Thus, citrate is transported to the cytosol for the production of acetyl-CoA through ATP citrate lyase [33]. In our study a similar abundance of isocitrate dehydrogenase and ATP-citrate lyase was observed in *X. dendrorhous* cultured in both carbon sources (Figure 3). In this sense, the increased expression of carotenogenic genes in a *X. dendrorhous* mutant was associated with the low content of ergosterol and fatty acids and the flux of acetyl-CoA for astaxanthin production [34].

### Availability of acetyl-CoA and its association with carotenogenesis

Acetyl-CoA is a key substrate for diverse cellular processes in the nucleus, mitochondria, cytosol and peroxisome. In the cell, acetyl-CoA is involved in several functions such as chromatin regulation, and it is a precursor for fatty acid and sterol biosynthesis [35]. In addition, acetyl-CoA is the end product of lipid degradation through β-oxidation and is essential for energy production in the TCA cycle and the synthesis of C4 metabolites through the glyoxylate cycle [36]. Furthermore, acetyl-CoA is also the precursor for diverse compounds such as waxes, flavonoids, carotenoids and isoprenoids [36].

During growth in glycolytic carbon sources, pyruvate is transformed into acetyl-CoA through the pyruvate dehydrogenase complex (PDA) in the mitochondria. In this sense, the *X. dendrorhous* α subunit of PDA showed greater abundance in glucose with a higher peak at the lag growth phase. In succinate, the α subunit of PDA showed a lower abundance with a peak during the late exponential phase, indicative of acetyl CoA synthesis through gluconeogenesis (Additional file 6: Figure S3A).

For some microorganisms, the pyruvate-acetaldehyde-acetate pathway generates acetyl-CoA, which is the main source of cytoplasmic acetyl-CoA in *S. cerevisiae* [35]. This pathway includes the enzyme pyruvate decarboxylase (PDC), acetaldehyde dehydrogenase (ALD) and acetyl-CoA synthase (ACS) [37]. In fungi, PDC is induced under aerobic conditions [37] and in the presence of glucose in *X. dendrorhous* [16]. Interesting, in the present study we observed a higher abundance of PDC in succinate growth (Additional file 6: Figure S3A).

ATP-citrate lyase (ACL) is an exclusive cytoplasmic source of acetyl-CoA in oleaginous yeast [35]. For *X. dendrorhous* the source of acetyl-CoA for carotenoid production could primarily be generated through an increased activity of ACL in the presence of O_2_ leading to carotenoid accumulation [38]. In the present study, a similar abundance of ACL was observed under both growth conditions (Figure 3). Therefore, the increased production of astaxanthin in succinate cultures is not directly associated with an increase of ACL abundance. On the other hand, the cytoplasmic enzyme ATP citrate synthase, which catalyzes the formation of acetyl-CoA, is primarily involved in the synthesis of fatty acids and sterols. The results presented herein showed a similar abundance of this enzyme in growth media containing either carbon source (Additional file 5: Figure S2B). Notably, in a recent study, high levels of succinate promoted an increase in cytosolic citrate synthase activity without inhibiting yeast growth [39].

In summary, these results suggest that the production of cytosolic acetyl-CoA is crucial for *X. dendrorhous* growth, which depends on the available carbon source. Thus, in glucose we observed a higher abundance of enzymes and metabolites of the pyruvate-acetaldehyde-acetate pathway derived from glycolysis and fermentation. In succinate, the low abundance of acetyl CoA carboxylase favors astaxanthin synthesis probably with reduced fatty acid content. Similar levels of the enzymes ATP citrate lyase and ATP-citrate synthase were observed during growth in both carbon sources, indicating the importance of cytoplasmic acetyl-CoA for generating fatty acids and astaxanthin.

### Oxidative stress and its association with the synthesis of astaxanthin in *X. dendrorhous*

Due to the antioxidant properties of carotenoids, the accumulation of these molecules is a survival strategy in various microorganisms [13]. Thus, oxidative stress stimulates the synthesis of carotenoids [40]. In addition to the degradation of cell membranes and lipids, ROS act as intracellular signaling molecules that activate cellular defense systems, thereby improving the resistance and adaptation of microorganisms in unfavorable environments [40,41]. Metabolic ROS production depends on the O_2_ concentration [42] therefore; ROS are endogenously generated in cells during respiration and β-oxidation [41]. In yeast, the increased amount of H_2_O_2_ generated during β-oxidation in peroxisome is neutralized with high levels of catalase [43]. However, the absence or low activity of this enzyme in *X. dendrorhous* has been reported [13], suggesting the important antioxidant role of carotenoids in this organism.

Cell antioxidant mechanisms also include non-enzymatic molecules such as glutathione and enzymatic systems such as SOD, catalase and glutathione peroxidase [31]. Apparently, *X. dendrorhous* exhibits low levels of these enzymatic systems [13]. Indeed, in the present study, only the mitochondrial MnSOD enzyme and some glutathione metabolic enzymes (Additional file 2: Table S1) were identified.

For the redox and stress response proteins, similar abundance pattern during carotenogenesis induction was observed. Thus, in glucose we observed the positive regulation of a monooxygenase (spot 5703), MnSOD (7108) and cytochrome P450 (spot 5315) (Additional file 3: Table S2A). Similarly, in succinate during the early exponential phase the monooxygenase (spot 4713) and cytochrome P450 (spot 5315) (Additional file 3: Table S2B) were increased. Within this group of proteins, specifically the P450 and monooxygenase could have a role in the induction of carotenogenesis independent of the carbon source. These two proteins are strongly associated with the biosynthesis of secondary metabolites, including carotenoids [16,18]. Thus these enzymes may perform auxiliary reactions for astaxanthin synthesis.

A stress indicator observed in the present study was trehalose accumulation. The synthesis of trehalose is considered as a defense mechanism against a variety of stress conditions including the presence of ROS [19,26], and the protective role of this sugar against ROS has been demonstrated in cell deficient in SOD [44]. The first reaction of the thehalose cycle is catalyzed through the glycolytic enzyme phosphoglucomutase [45]. Interestingly, in the present study, this enzyme exhibited both glucose and succinate abundance coincident with carotenogenesis induction (Additional file 3: Table S2A and B). Correspondingly, trehalose was the most abundant metabolite observed in the present study. When the amount of thehalose (Additional file 4: Table S3) was compared between the two carbon sources, a significant increase was observed in succinate during the late exponential phase and the stationary phase.

Related to the stress response an increase in several carbohydrate metabolism enzymes has been found, which might redirect the carbon flux to generate antioxidant substrates or activate the production of cell protective substances. For instance, when stress is induced in *H. pluvialis*, a transient increase in several glycolysis and PP pathway proteins was observed [20], indicating that the early response to stress includes enzymatic defense processes and affects central metabolic pathways, leading to the cellular accumulation of astaxanthin. In the present study, GAPDH and phosphoglucomutase were positively regulated during the carotenogenesis induction (Additional file 3: Table S2 (A and B)). Interesting, in *S. cerevisiae* three isoforms of GAPDH associated with glycolysis, gluconeogenesis, and the stress response were observed [46]. In our study, GAPDH was identified in multiple points which indicate a probable post-translational regulation of this enzyme according to the carbon source (Additional file 2: Table S1).

In *S. cerevisiae* a common, cross reaction response to various stress conditions has been observed [45], suggesting that various stress situations generate high oxidant conditions, leading to stress responses in cells. A stress core has been identified in *S. cerevisiae*, *S. pombe* and *C. albicans* [47-49]. The results of the present study, suggest that in *X. dendrorhous*, the antioxidant core during carotenogenesis induction in glucose and succinate includes a monooxygenase, a cytochrome P450 enzyme, phosphoglucomutase and GAPDH.

In summary, our results clearly support a relationship between the synthesis of astaxanthin and response to stress in *X. dendrorhous*. First, several redox- and stress-related proteins were identified, and the modulation in their abundance during the induction of carotenogenesis were determined. These proteins have been previously identified in other proteomic studies associated with different types of stress responses in several microorganisms. Second, the induction of the carotenoid synthesis was consistent with the positive regulation of phosphoglucomutase, which initiates synthesis of trehalose that is a major metabolite identified in both carbon sources as a stress indicator. Third, only one typical antioxidant enzyme, MnSOD, was identified. Therefore, these results suggest that during aerobic growth, the concerted expression of stress-related proteins induces carotenogenesis as a protective mechanism in *X. dendrorhous*.

### Carotenogenesis and related pathways in *X. dendrorhous*

In *X. dendrorhous*, IPP, a general precursor of all isoprenoids, is produced through the MVA pathway (Figure 5). This pathway is also the precursor for ergosterol formation. Therefore, the functioning of the cell signaling processes, the maintenance of membrane integrity, prenylation and protein glycosylation depend on the MVA pathway [50].

The main regulatory enzyme of the MVA pathway is HMGR [50]. Recently we observed that in *X. dendrorhous* the gene expression of this enzyme is likely regulated through ergosterol via negative feedback [3]. Similarly, the levels of GPP, FPP and GGPP regulate MK, another MVA pathway enzyme [50]. In the present study, the amount of MK (spot 4609) in succinate was significantly higher than that in glucose (Table 1). This result is consistent with observations in succinate cultures, where a high level of TCA intermediates associated with the presence of an increased amount of available substrate (acetyl-CoA) was observed.

Concerning the specific carotenogenic pathway in *X. dendrorhous*, glucose exhaustion has been correlated with the high levels of carotenogenic mRNAs [18]. In contrast, the transcriptional regulation in succinate remains unclear; indeed, the carotenogenic genes are constitutively expressed [18]. In the present study, the abundance of only a single carotenogenic enzyme, the phytoene/squalene synthase (spot 4515), was observed during the late exponential growth phase when the yeast was cultured in glucose (Additional file 3: Table S2A). For the other carotenogenic enzymes, no clear relation with the reported transcriptional regulation profile was observed.

Additionally, the enzymes involved in steps up-stream to lycopene formation were abundant in glucose culture. However, the enzymes involved in the last step of carotenogenesis, CPR (spot 7311) and astaxanthin synthase (spot 7501) (Table 1), showed significant abundance in succinate culture, suggesting a post-translational regulation mechanism.

## Conclusions

In the present study, comprehensive analyses of the proteins and metabolites profile, produced in yeast cultured with fermentable (glucose) and non-fermentable (succinate) carbon sources were performed. The utilization of glucose or succinate as a sole carbon source showed clear distinguishable differences in principal and related metabolic pathways. The main differences observed corresponded to the enhancement of the glycolysis and PP pathways in glucose, and TCA and glyoxylate cycles in succinate.

Regulation at the protein level was associated with astaxanthin biosynthesis. First, under fermentative conditions, the cell responds to ensure the acetyl-coA supply through various metabolic alternatives. Second, the concerted positive regulation of redox- and stress-related proteins (monooxygenase, cytochrome P450, phosphoglucomutase and GAPDH) is correlated with carotenogenesis induction. Third, trehalose accumulation, associated with oxidative stress, influences carotenogenesis. Fourth, the differential protein abundance of carotenogenic enzymes is not correlated with the carotenogenic transcript pattern; indeed, for late carotenogenic enzymes, post-translational regulation is likely involved.

Moreover, these results suggest that astaxanthin is an alternative mechanism in response to cellular or environmental stress conditions in *X. dendrorhous*.

## Methods

### Yeast strain and culture conditions

MM supplemented with 2% of glucose or succinate as a carbon source was used for growth of the wild-type *X. dendrorhous* strain UCD 67–385 [17]. The main culture (250 ml) was inoculated with 2.5 ml of an exponential phase culture and grown at 22°C with constant swirling at 120 rpm. For data analysis, samples were collected at different time points (the lag, early exponential, late exponential and stationary growth phases) (Figure 1). For proteomic studies, cells were harvested through centrifugation at 5,000 × g for 10 min at 4°C. The pellet was washed twice with ice-cold water and centrifuged at 5,000 × g for 10 min at 4°C. Subsequently, the washed pellet was frozen in liquid nitrogen and stored at −80°C. The cell density was determined optically at 560 nm using a spectrophotometer and gravimetrically by measuring the cell dry weight.

### Preparation of the proteins extracts

Proteins were extracted as previously described [17]. Briefly, an equal volume (approximately 500 μl) of glass beads was added to the lyophilized cells. The cells were disrupted for 30 s at 4.5 m/s in a RiboLyzer (Hybaid-AGS, Heidelberg, Germany) and chilled on ice for 1 min. Subsequently, 500 μl of lysis buffer (100 mM sodium bicarbonate, pH 8.8, 0.5% Triton X-100, 1 mM phenylmethylsulfonyl fluoride [PMSF] and protease inhibitors [Roche, Mannheim, Germany]) was added and the samples were incubated for 15 min on ice. The cells were disrupted five times in a RiboLyzer and chilled on ice for 1 min between vortexing steps. Subsequently, the sample was centrifuged at 15,000 rpm for 20 min at 4°C and the supernatant was collected in 1.5-ml tubes. The protein extracts were incubated for 1 h at 4°C in a 10% v/v DNase-RNase solution (0.5 M Tris–HCl, pH 7.0, 0.5 M MgCl_2_, 100 μg/ml RNase A [Boehringer Mannheim, Germany] containing 2 μl DNase I [Boehringer Mannheim]) and the final volume was adjusted to 2.5 ml with deionized water. Next, 200 μl of 0.5 M Tris (pH 6.8) and 20 μl of 1 M dithiothreitol (DTT) were added, and the mixture was incubated for 30 min at room temperature (RT). Subsequently, 600 μl of water-saturated phenol was added and the suspension was vigorously shaken for 30 min. The phenol phase was removed and transferred into a fresh reaction tube, followed by the addition of 20 μl of 1 M DTT and 30 μl 8 M ammonium acetate. After 30 min, 2 ml of cold methanol was added, and incubated over night at −20°C to precipitate the proteins. Subsequently, the precipitate was centrifuged at 13,000 rpm at 4°C for 30 min and the pellet was washed twice with 70% (v/v) cold ethanol at −20°C. The proteins were solubilized in 200 μl of rehydration buffer (8 M urea, 2 M thiourea, 2% [w/v] 3[(3-cholamidopropyl)dimethylammonio]-1-propanesulphonate [CHAPS], 0.01% [w/v] bromophenol blue) and stored at −80°C. The protein concentration was measured using a Bradford- based protein assay (Bio-Rad, Hercules, CA) with bovine serum albumin as a standard.

### 2D electrophoresis

The volume of the proteins extracts was adjusted to a final protein concentration of 500 μg in 340 μl of rehydration buffer. Subsequently, 1% DTT and 2% immobilized pH gradient (IPG) buffer pH 3–10 (Amersham Biosciences, Freiburg, Germany) were added. The protein sample was loaded onto 17- cm, non- linear pH 3–10 IEF isoelectric focusing strips (Immobiline DryStrip, Amersham Biosciences). IEF was performed using a IPGphor™ system (Amersham Biosciences) with the following program: 10 h at 20°C, 12 h at 30 V, 1 h at 500 V, 8 h at 1,000 V and 10 h at 8,000 V.

The strips were equilibrated for 15 min in 10 ml of equilibration solution (0.375 M Tris–HCl, pH 8.8, 6 M urea, 20% [v/v] glycerol and 2% [w/v] SDS), with 2% (w/v) DTT and for 15 min in 10 ml of equilibration solution containing 2% (w/v) iodoacetamide. Subsequently, the strip was transferred onto a 10% SDS-PAGE gel for second-dimension electrophoresis using a Bio-Rad Protean II system with 30 W per gel. The gels were subsequently stained with Coomassie brilliant blue.

### Image analysis

The stained gels were scanned and analyzed using PDQuest software (version 7.1.1, Bio-Rad) as previously described [17]. Briefly, the normalized spots intensities were used to calculate the mean and standard deviation in using MatchSets software tool. The average -fold differences in protein abundance were calculated as the ratio between the relative abundance levels at different sampling phases in succinate/glucose. A *t*-test with Benjamini-Hochberg (p < 0.05) false discovery rate (FDR) correction for multiple testing was performed for the statistical analyses of the changes in protein abundance [51].

### MS analyses and database searches

For trypsin digestion, Coomassie-stained protein spots were excised from the gel and washed with 150 μl of 50% acetonitrile (ACN) for 5 min, followed by an additional wash with 150 μl of 50 mM NH_4_HCO_3_ and 50% ACN for 30 min, and a final wash with 150 μl of 10 mM NH_4_HCO_3_ for 30 min with stirring at RT. After removing the supernatant, the gel was completely dried at RT. For digestion, 15 μl of a 2.5 mg/ml trypsin solution (Promega, Madison, WI) in 10 mM NH_4_HCO_3_ was added and the solution was incubated at 37°C overnight.

Mass-spectrometry was performed using the Ultraflex™ (Bruker, Bremen, Germany) MALDI-TOF-MS. The sample positions on an Anchor-Chip™ (Bruker, Bremen, Germany) were pre-treated with 3 μl of α-cyano-4-hydroxy cinnamic acid and subsequently dried. Then, 1 μl of trypsin-digested sample was applied and dried. The spectra data were analyzed using FlexAnalysis software (Bruker-Daltonics). The peptide mass fingerprints (PMF) were interpreted using the Mascot search engine run on local server (Matrix Science, London, UK). For protein identification the NCBI, Swiss-Prot and a local database with partial sequences of *X. dendrorhous* contigs were employed. The following parameters were applied for data mining: taxonomic category, fungi; no MW/pI restrictions; enzyme: trypsin; missed cleavages: 1; peptide tolerance: 150 ppm; mass value: MH^+^ mono isotopic; fixed modifications: carbamido methyl; and variable modifications: methionine oxidation. A protein was only considered to be significant if it was identified at least twice from the same position in independent gels, the MOWSE score was higher than 50 (p < 0.05) and was the same in two of the three analyzed databases. For contig identification, the significant Mascot score was higher than 40 (p < 0.05). The identified proteins were classified according to the Kyoto Encyclopedia of Genes and Genomes (KEGG) pathway database [52].

### Metabolite extraction and derivatization

For each extraction three biological replicates were employed following the sample treatment [53] with some modifications. Rapidly in ice, the mix of 2 ml of culture and 2 ml of 60% v/v methanol at −20°C was incubated at −20°C for 15 min. After 5,500 rpm centrifugation at −15°C for 15 min, the supernatant was removed. Subsequently, the pellet was washed with 5 ml of 60% v/v methanol at −20°C and centrifuged at −15°C for 5 min at 5,500 rpm. The final pellet was freeze-dried and instantly frozen in liquid nitrogen until further use. The pellet weight was determined prior to metabolite extraction.

The intracellular metabolite extraction and all derivatization steps were performed as previously described [54]. Briefly, 1 ml of 80% methanol containing 1 μM Ribitol (internal standard) was added to a total of 3–5 mg freeze-dried sample of the yeast in 1.5-ml screw cap tubes with 0.5 g acid-washed glass beads (Sigma-Aldrich). Immediately after adding methanol, the cells were disrupted using a FastPrep™ (Qbiogene, Heidelberg, Germany) three times at 6.5 ms^−1^ for 45 s. Metabolite extraction was enhanced through incubation at 70°C for 15 min with 1,400 rpm agitation in a Thermomixer (Eppendorf, Hamburg, Germany). After 20 min centrifugation at 18,500 g at RT, the clear supernatant was evaporated to total dryness under a nitrogen stream. For the derivatization of the medium compounds, methoximation of carbonyl moieties was performed at 37°C for 90 min under constant stirring using 75 μl of a 20 mg solution of methoxylamine hydrochloride in pyridine. Acidic protons were protected with trimethylsilyl groups through treatment with 50 μl N-methyl-N-[trimethylsilyl]trifluoroacetamide at 37°C for 30 min.

### GC-MS

Sample volumes of 1 μl were analyzed using a TraceGC gas chromatograph coupled to a PolarisQ ion trap mass spectrometer equipped with an AS2000 auto sampler (Thermo Finnigan, Dreieich, Germany). The derivatized metabolites were evaporated at 250°C in splitless mode and separated on a 30 m × 0.25 mm VF-5 MS capillary column (Varian, Darmstadt, Germany). The helium carrier gas was set to a constant flow of 1 ml/min. The interface temperature was adjusted to 250°C and the ion source was set to 200°C. After 3 min constant heating at 80°C, the oven temperature was raised in increments of 5°C/min to 325°C where it was held for 5 min. Mass spectra were recorded at 2 scans/s with a scanning range of 50–550 m/z. Relative levels of selected metabolites were determined through the integration of the peak areas of selective ions using the Xcalibur 2.0 software (Thermo Electron, Dreieich, Germany). Relative response ratios were calculated after normalizing the respective peak areas to the peak area of the internal standard and dividing the value by the weight of the extracted sample. For data analyses, three independent biologic replicates were used. Metabolites were identified through comparison with purified standards (Sigma) and the NIST 98 database (NIST, Gaithersburg, MD). The identified metabolites were classified based on the KEGG database [52]. The normalized log_2_ ratios were used to graphically represent the data. For the metabolite mapping the average -fold differences in abundance was calculated as the ratio between the relative abundance levels at different sampling phases in succinate/glucose. A *t*-test with Benjamini-Hochberg (p < 0.05) false discovery rate (FDR) correction for multiple testing was performed for the statistical analyses of the changes in metabolite abundance [51].

### Carotenoid extraction and HPLC analysis

Total carotenoids were extracted from cellular pellets using an acetone extraction method [55]. Carotenoids were quantified by absorbance at 465 nm with an absorption coefficient of A1% = 2,100. The analyses were performed in triplicate and the pigments were normalized relative to the dry weight of the yeast. Carotenoids were separated through RP-HPLC using a reverse phase RP-18 Lichrocart 125–4 (Merck) column and acetonitrile: methanol; isopropanol (85: 10: 5 v/v) mobile phase with a 1 ml/min flow rate under isocratic conditions. The elution spectra were recovered using a Shimadzu SPD-M10A diode array detector. Carotenoids were identified based on the spectra and retention time according to standards.

## Additional files

Additional file 1: Figure S1. Proteomic profile of *X. dendrorhous* during growth. A) Glucose, B) Succinate. The image was obtained using PDQuest software ver. 7.1.1. For each carbon source employed during the yeast growth, each 2D-gel represents one of the triplicates obtained for the protein analysis. The proteins were separated using isoelectric focusing in a gradient of pH 3(left) to pH 10 (right), followed by sodium dodecyl sulfate-polyacrylamide gel electrophoresis. The gels were stained with Coomassie blue. Additional file 2: Table S1.
*X. dendrorhous* proteins identified by MALDI-TOF MS. This table lists all MS-identified proteins separated through 2D electrophoresis. Additional file 3: Table S2.
*X. dendrorhous* proteins differential abundance when cultured in glucose or succinate. Differentially regulated proteins observed during *X. dendrorhous* growth are shown. Additional file 4: Table S3. Relative changes in the yeast metabolite abundance when cultured in succinate versus glucose. Differentially regulated metabolites observed during *X. dendrorhous* growth are shown. Additional file 5: Figure S2. Protein and metabolite levels in sugar and lipid metabolism. According to the carbon source the mean value and SD of abundance for each protein, and the response ratio for each metabolite were normalized to a value of 100. Each column graphic represents the growth phases studied (from the left to the right: lag, early exponential, late exponential and stationary). In the column charts a color code was included to differentiate proteins and metabolites found when *X. dendrorhous* was cultured with the different carbon sources: proteins in glucose (orange), proteins in succinate (cyan), metabolites in glucose (red) and metabolites in succinate (green). The pathways were adapted from the KEGG database. In the metabolic pathways, names are written in a color letters: metabolites (black), sugar proteins (blue), lipid metabolism proteins (brown). An asterisk beside the protein name and in the multiple column charts indicates that the protein probably suffers post-translational modifications as it was identified in multiple spots. Statistical significant differences between samples from different carbon sources at the same growth phase are represented as stars (*t*-test p < 0.02) and triangles (Benjamini-Hochberg correction p < 0.05). Abbreviations: P: phosphate, DHAP: dihydroxy acetone phosphate, UDP: uridine diphosphate, P: phosphate, ACP: acyl transport protein, CDP: cytidine diphosphate, FAS: fatty acid synthase. Additional file 6: Figure S3. Protein and metabolite levels in the oxidative phosphorylation and pyruvate metabolism. According to the carbon source the mean value and SD of abundance for each protein, and the response ratio for each metabolite were normalized to a value of 100. Each column graphic represents the growth phases studied (from left to the right: lag, early exponential, late exponential and stationary). In the column charts a color code was included to differentiate proteins and metabolites found when *X. dendrorhous* was cultured with the different carbon sources: proteins in glucose (orange), proteins in succinate (cyan), metabolites in glucose (red) and metabolites in succinate (green). The pathways were adapted from the KEGG database. In the metabolic pathways, names are written in a color letters: metabolites (black), oxidative phosphorylation proteins (blue) and pyruvate metabolism proteins (purple). An asterisk beside the protein name and in the multiple column charts indicates that the protein probably suffers post-translational modifications as it was identified in multiple spots. Statistical significant differences between samples from different carbon sources at the same growth phase are represented as stars (*t*-test p < 0.02) and triangles (Benjamini-Hochberg correction p < 0.05). Abbreviations: FMN: flavin mononucleotide, ThPP: thiamine diphosphate.

## Abbreviations

*X. dendrorhous*: *Xanthophyllomyces dendrorhous*
MM: Minimal medium
TCA: Tricarboxylic acid cycle
PP pathway: Pentose phosphate pathway
IPP: Isopentenyl pyrophosphate
GGPP: Geranyl geranyl pyrophosphate
GGPS: Geranyl geranyl pyrophosphate synthase
RP-HPLC: Reverse phase- high pressure liquid chromatography
GAPDH: Glyceraldehyde 3-phosphate dehydrogenase
ADH: Alcohol dehydrogenase
ROS: Reactive oxygen species
SOD: Superoxide dismutase
HMGR: 3-hydroxy-3-methyl-glutaryl-CoA reductase
MK: Mevalonate kinase
CPR: Cytochrome P450 reductase
FAS: Fatty acid synthase
NADPH: Nicotinamide adenine dinucleotide phosphate
