# Supplementary figures and images for: Proteomic and metabolomic analysis of the carotenogenic yeast Xanthophyllomyces dendrorhous using different carbon sources
Source: BMC Genomics. 2015 Apr 12;16(1):289. doi: 10.1186/s12864-015-1484-6 (PMC4404605; doi:10.1186/s12864-015-1484-6)

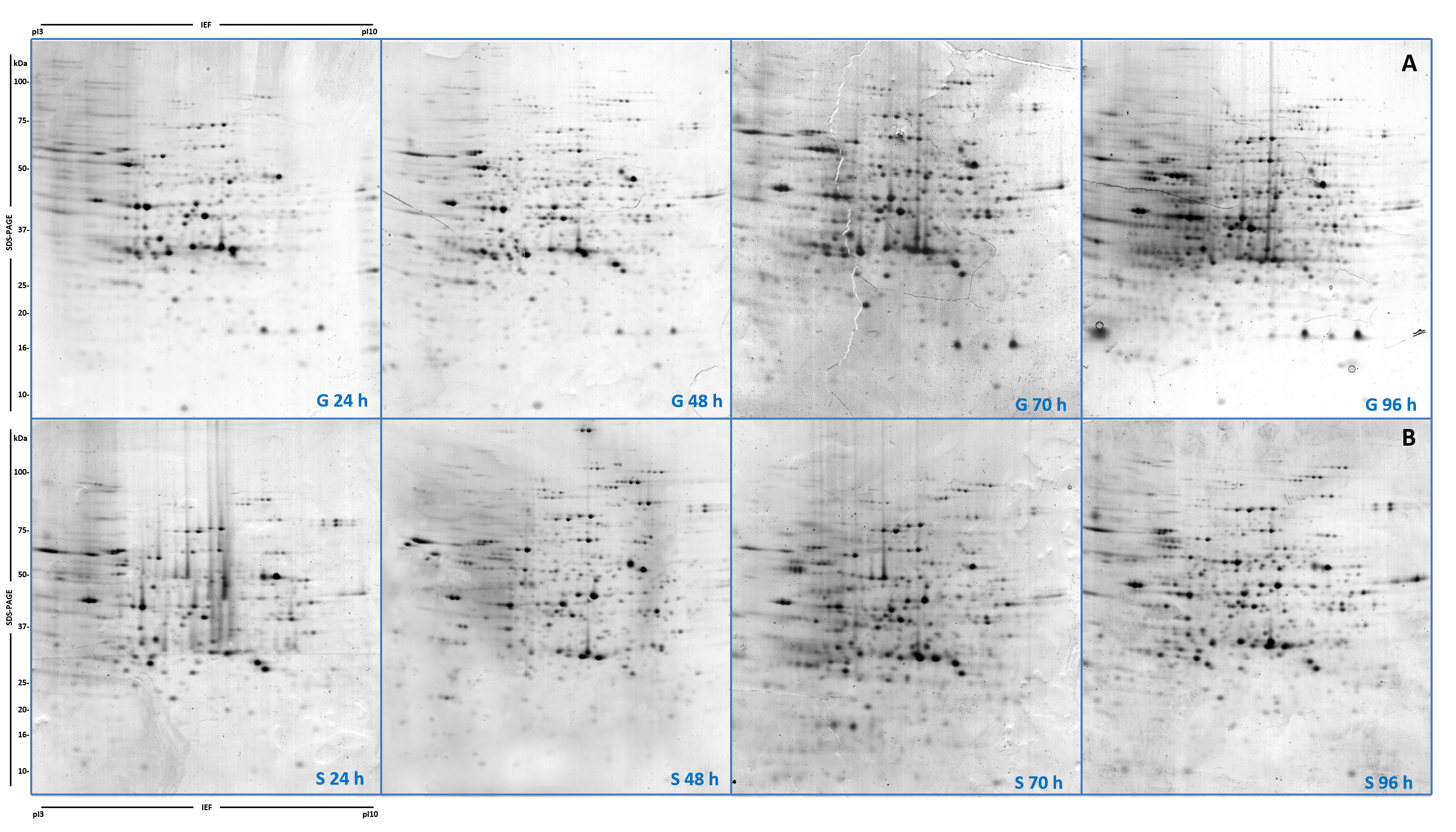

Supplement: Additional file 1: Figure S1. — Proteomic profile of X. dendrorhous during growth. A) Glucose, B) Succinate. The image was obtained using PDQuest software ver. 7.1.1. For each carbon source employed during the yeast growth, each 2D-gel represents one of the triplicates obtained for the protein analysis. The proteins were separated using isoelectric focusing in a gradient of pH 3(left) to pH 10 (right), followed by sodium dodecyl sulfate-polyacrylamide gel electrophoresis. The gels were stained with Coomassie blue. [file 12864_2015_1484_MOESM1_ESM.tiff]

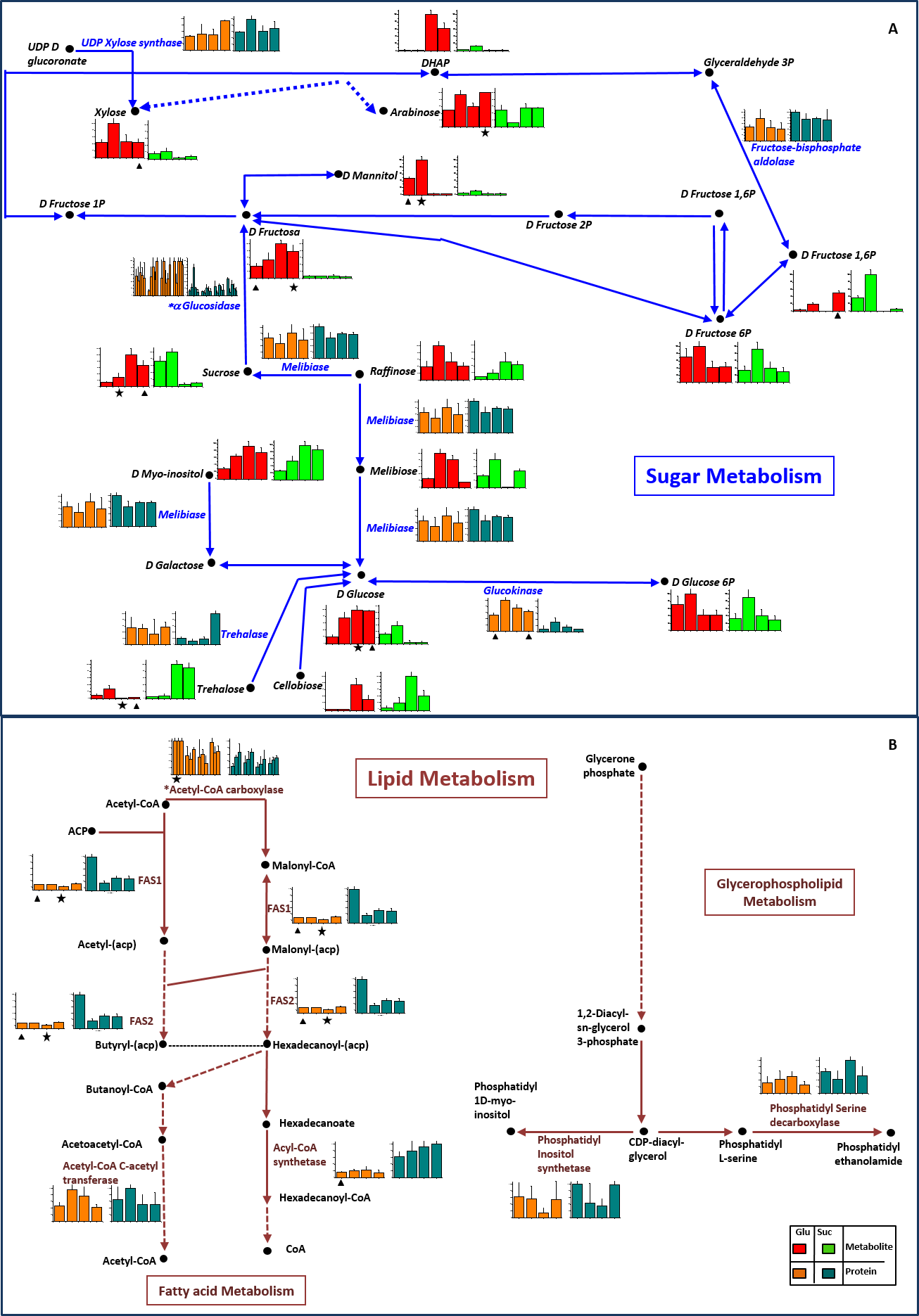

Supplement: Additional file 5: Figure S2. — Protein and metabolite levels in sugar and lipid metabolism. According to the carbon source the mean value and SD of abundance for each protein, and the response ratio for each metabolite were normalized to a value of 100. Each column graphic represents the growth phases studied (from the left to the right: lag, early exponential, late exponential and stationary). In the column charts a color code was included to differentiate proteins and metabolites found when X. dendrorhous was cultured with the different carbon sources: proteins in glucose (orange), proteins in succinate (cyan), metabolites in glucose (red) and metabolites in succinate (green). The pathways were adapted from the KEGG database. In the metabolic pathways, names are written in a color letters: metabolites (black), sugar proteins (blue), lipid metabolism proteins (brown). An asterisk beside the protein name and in the multiple column charts indicates that the protein probably suffers post-translational modifications as it was identified in multiple spots. Statistical significant differences between samples from different carbon sources at the same growth phase are represented as stars (t-test p < 0.02) and triangles (Benjamini-Hochberg correction p < 0.05). Abbreviations: P: phosphate, DHAP: dihydroxy acetone phosphate, UDP: uridine diphosphate, P: phosphate, ACP: acyl transport protein, CDP: cytidine diphosphate, FAS: fatty acid synthase. [file 12864_2015_1484_MOESM5_ESM.tiff]

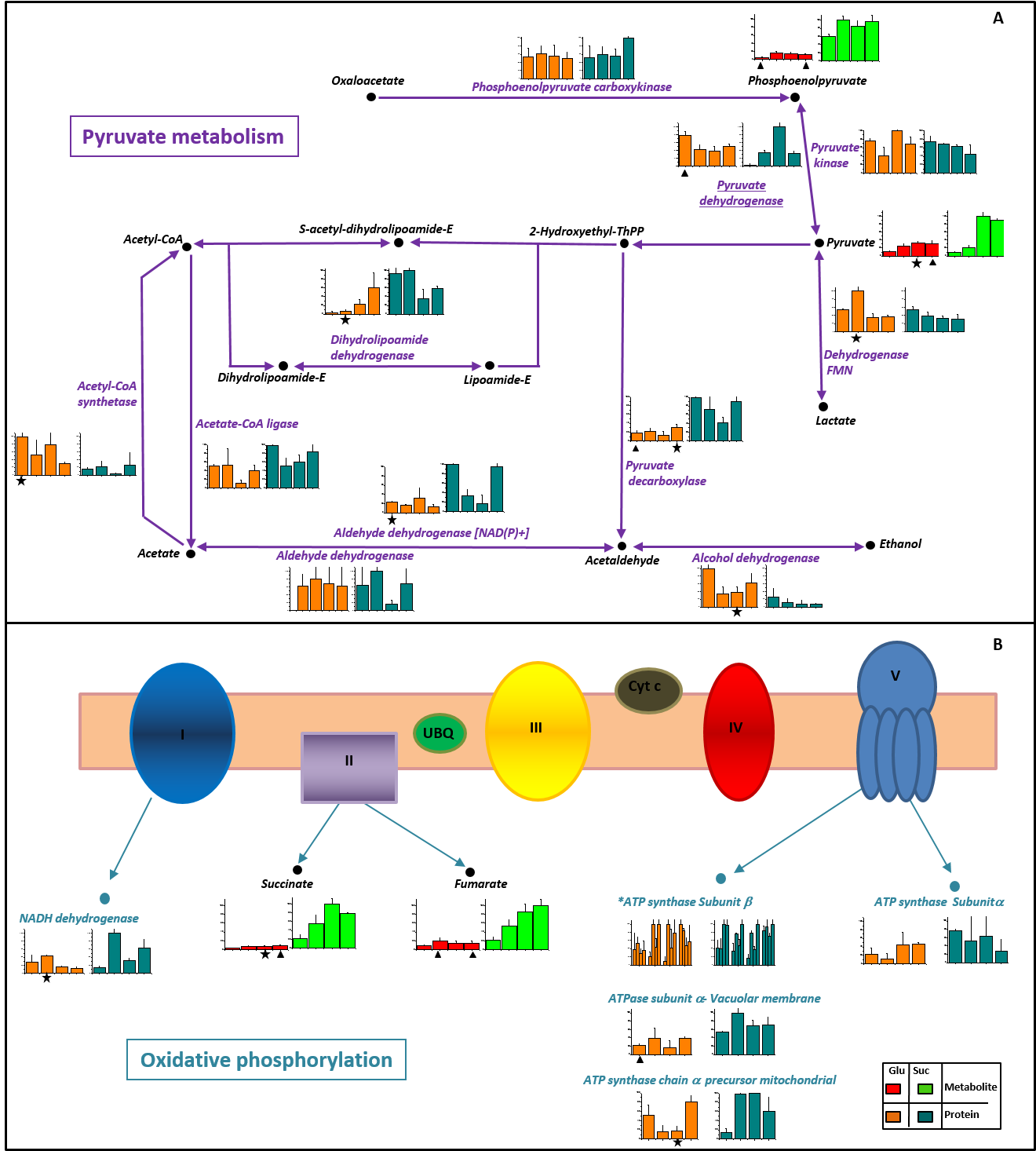

Supplement: Additional file 6: Figure S3. — Protein and metabolite levels in the oxidative phosphorylation and pyruvate metabolism. According to the carbon source the mean value and SD of abundance for each protein, and the response ratio for each metabolite were normalized to a value of 100. Each column graphic represents the growth phases studied (from left to the right: lag, early exponential, late exponential and stationary). In the column charts a color code was included to differentiate proteins and metabolites found when X. dendrorhous was cultured with the different carbon sources: proteins in glucose (orange), proteins in succinate (cyan), metabolites in glucose (red) and metabolites in succinate (green). The pathways were adapted from the KEGG database. In the metabolic pathways, names are written in a color letters: metabolites (black), oxidative phosphorylation proteins (blue) and pyruvate metabolism proteins (purple). An asterisk beside the protein name and in the multiple column charts indicates that the protein probably suffers post-translational modifications as it was identified in multiple spots. Statistical significant differences between samples from different carbon sources at the same growth phase are represented as stars (t-test p < 0.02) and triangles (Benjamini-Hochberg correction p < 0.05). Abbreviations: FMN: flavin mononucleotide, ThPP: thiamine diphosphate. [file 12864_2015_1484_MOESM6_ESM.tiff]
