# Supplementary material for: Proteomic and metabolomic analysis of the carotenogenic yeast Xanthophyllomyces dendrorhous using different carbon sources
Source: BMC Genomics. 2015 Apr 12;16(1):289. doi: 10.1186/s12864-015-1484-6 (PMC4404605; doi:10.1186/s12864-015-1484-6)
Supplement: Additional file 2: Table S1. — X. dendrorhous proteins identified by MALDI-TOF MS. This table lists all MS-identified proteins separated through 2D electrophoresis. [file 12864_2015_1484_MOESM2_ESM.docx]

**Table S1. *X. dendrorhous* proteins identified by MALDI-TOF MS.**

| N° | ID | Score | Peptide | Coverage % | Assignment | CC | Specie | Spot # | Mr/pI | |
| --- | --- | --- | --- | --- | --- | --- | --- | --- | --- | --- |
|  |  |  |  |  |  |  |  |  | Theoretical | Experimental |
|  |  |  |  |  | **Cellular Processes: Transport and Motor Proteins** |  |  |  |  |  |
| 1 | 19112071 | 60 | 5 | 5 | Putative coatomer subunit alpha | M | *Schizosaccharomyces pombe* | 6806 | 137.42/5.76 | 122.57/6.15 |
|  |  | 57 | 5 | 5 |  |  |  | 6813 |  | 122.55/6.23 |
|  |  | 46 | 4 | 3 |  |  |  | 6818 |  | 122.54/6.24 |
| 2 | 121792722 | 65 | 8 | 9 | Myosin -associated protein | - | *Chaetomium globosum* | 8703 | 107.26/8.01 | 107.21/7.70 |
|  |  | 64 | 9 | 10 |  |  |  | 8711 |  | 107.26/8.30 |
| 3 | 46099667 | 67 | 8 | 10 | Golgi transport protein | M | *Ustilago maydis* | 5719 | 124.27/6.17 | 101.09/6.07 |
|  |  | 52 | 5 | 7 |  |  |  | 5728 |  | 101.94/5.92 |
|  |  | 73 | 8 | 10 |  |  |  | 6703 |  | 100.63/6.14 |
| 4 | 151941363 | 52 | 12 | 9 | Myosin V | - | *Saccharomyces cerevisiae* | 1815 | 169.94/7.31 | 109.52/5.32 |
|  |  | 62 | 12 | 11 |  |  |  | 1816 |  | 109.41/5.31 |
|  |  | 56 | 11 | 11 |  |  |  | 1817 |  | 109.40/5.31 |
| 5 | 70997653 | 90 | 7 | 12 | Sec1 family superfamily | - | *Aspergillus fumigatus* | 6722 | 76.29/8.03 | 86.39/6.23 |
| 6 | 156063588 | 57 | 12 | 16 | SS1G_01912 | - | *Sclerotinia sclerotiorum* | 2703 | 97.47/9.14 | 76.08/5.34 |
|  |  | 55 | 11 | 15 |  |  |  | 2707 |  | 76.53/5.33 |
|  |  | 64 | 13 | 17 |  |  |  | 2712 |  | 76.27/5.35 |
| 7 | 190407814 | 68 | 14 | 15 | ABC transporter of long-chain fatty acids | M | *Saccharomyces cerevisiae* | 6613 | 100.39/9.53 | 61.94/6.25 |
| 8 | 49650784 | 56 | 6 | 7 | KIP1 kinesin- related protein | - | *Yarrowia lipolytica* | 7403 | 109.59/8.16 | 45.58/6.39 |
|  |  | 51 | 7 | 10 |  |  |  | 7408 |  | 45.36/6.46 |
| 9 | 85110027 | 56 | 4 | 16 | ADP,ATP carrier protein | M | *Neurospora crassa* | 8412 | 33.98/9.84 | 45.89/7.59 |
| 10 | 50547781 | 54 | 6 | 12 | Golgi transport component | - | *Yarrowia lipolytica* | 5520 | 78.90/5.68 | 52.33/6.03 |
| 11 | 1703157 | 145 | 10 | 46 | Actin | - | *Phaffia rhodozyma* | 3305 | 41.93/5.38 | 41.94/5.38 |
| 12 | 164426677 | 64 | 20 | 10 | Fimbrin | - | *Neurospora crassa* | 5201 | 61.50/5.77 | 34.00/5.81 |
| 13 | 83770850 | 68 | 5 | 19 | Mitochondrial oxodicarboxylate carrier | M | *Aspergillus oryzae* | 3330ª | 31.32/9.96 | 40.42/5.40 |
| 14 | 116191247 | 68 | 8 | 25 | Siderophore-iron transmembrane transporter | - | *Chaetomium globosum* | 5519 | 45.70/8.96 | 52.53/5.85 |
| 15 | 74582611 | 65 | 8 | 43 | Vacuolar protein-sorting-associated protein 25 | - | *Schizosaccharomyces pombe* | 7804 | 20.83/7.71 | 108.56/6.27 |
| 16 | 74585578 | 74 | 13 | 9 | Hypothetical protein NUM1 | - | *Candida albicans* | 8301 | 173.08/5.42 | 41.34/6.84 |
| 17 | 74693759 | 52 | 3 | 11 | AEL126Wp. Metal ion transmembrane transporter activity | M | *Ashbya gossypii* | 3330b | 43.60/5.78 | 40.42/5.40 |
| 18 | s6_c1087_p2 | 77 | 6 | 25 | Porin 3, putative | M | *X. dendrorhous* | 9210 | 30.78/9.13 | 34.40/9.70 |
| 19 | 6934202 | 52 | 5 | 14 | Beta-tubulin 1 | - | *Spiromyces minutus.* | 6603ª | 43.17/5.37 | 67.15/6.15 |
| 20 | cDNA6_c9170 | 70 | 6 | 14 | Alpha-tubulin | - | *X. dendrorhous* | 2503 | 45.45/4.89 | 63.80/5.30 |
| 21 | cDNA5_c9584 | 53 | 9 | 19 | Vacuolar membrane protein | M | *X. dendrorhous* | 6413 | 61.71/9.31 | 45.50/6.30 |
| 22 | cDNA5_c8096 | 51 | 4 | 61 | Chitin synthase export chaperone | M | *X. dendrorhous* | 1004 | 8.80/9.99 | 10.60/5.30 |
| 23 | cDNA5_c9597 | 59 | 11 | 23 | Coenzyme A transporter | - | *X. dendrorhous* | 4405 | 49.55/10.19 | 49.00/5.60 |
| 24 | cDNA5_c2239 | 94 | 8 | 10 | Related to neutral amino acid permease | M | *X. dendrorhous* | 7807 | 100.9/10.05 | 109.30/6.30 |
|  |  |  |  |  | **Environmental Information Processing: Signal Transduction** |  |  |  |  |  |
| 25 | 226287371 | 50 | 6 | 15 | Negative regulator of the PHO system. Ser-thr kinase | - | *Paracoccidioides brasiliensis* | 5515 | 53.42/7.03 | 53.61/6.10 |
| 26 | 130710 | 60 | 5 | 12 | Serine/threonine-protein phosphatase PP1-1 | - | *Saccharomyces cerevisiae* | 3717 | 35.53/5.23 | 107.99/5.42 |
|  |  | 63 | 4 | 10 |  |  |  | 3718 |  | 108.00/5.39 |
|  |  | 65 | 4 | 15 |  |  |  | 3808 |  | 108.15/5.49 |
|  |  | 49 | 5 | 19 |  |  |  | 3814 |  | 108.14/5.52 |
|  |  | 58 | 8 | 26 |  |  |  | 3815 |  | 108.11/5.45 |
|  |  | 54 | 6 | 14 |  |  |  | 3816 |  | 108.19/5.38 |
| 27 | 74626964 | 68 | 7 | 14 | Nucleotide phosphodiesterase | - | *Candida albicans* | 5724 | 66.07/5.87 | 79.30/6.10 |
| 28 | 6324708 | 66 | 7 | 23 | Rho-GTPase-activating protein BAG7 | - | *Saccharomyces cerevisiae* | 8605 | 46.30/6.66 | 57.74/8.21 |
| 29 | cDNA5_c7972 | 100 | 8 | 45 | 14-3-3. DNA damage checkpoint protein | - | *X. dendrorhous* | 126 | 21.03/5.45 | 29.20/4.65 |
|  |  | 66 | 5 | 30 |  | - |  | 127 |  | 29.10/4.56 |
|  |  | 51 | 4 | 23 |  | - |  | 128 |  | 29.00/4.30 |
| 30 | 74670489 | 76 | 15 | 10 | GTPase activating protein | - | *Aspergillus fumigatus* | 8502 | 142.88/7.38 | 59.96/6.90 |
| 31 | 74683137 | 58 | 10 | 17 | Protein kinase | - | *Cryptococcus neoformans* | 2321ª | 64.11/5.40 | 40.04/5.35 |
|  |  |  |  |  | **Genetic Information Processing** |  |  |  |  |  |
| 32 | 121787153 | 62 | 6 | 28 | Ribosomal_L15 | - | *Chaetomium globosum* | 9206 | 32.84/10.21 | 35.60/10.10 |
| 33 | 70989247 | 55 | 4 | 27 | Alkaline serine protease | - | *Aspergillus fumigatus* | 5207 | 13.60/9.72 | 34.96/6.05 |
| 34 | 68469184 | 73 | 9 | 19 | 60S ribosomal protein L5 | - | *Candida albicans* | 7304 | 34.48/7.08 | 40.18/6.38 |
| 35 | 50419303 | 73 | 8 | 26 | Protein PXR1 | - | *Debaryomyces hansenii* | 8104 | 37.01/9.87 | 22.46/7.28 |
| 36 | 119397404 | 64 | 6 | 16 | Eukaryotic translation initiation factor 3 subunit H | - | *Aspergillus clavatus* | 5417 | 41.26/5.96 | 47.08/5.81 |
| 37 | 156057585 | 51 | 4 | 24 | GTP-binding nuclear protein GSP1/Ran | - | *Sclerotinia sclerotiorum* | 5105 | 24.72/6.53 | 29.04/6.04 |
| 38 | cDNA5_c9778 | 169 | 18 | 45 | Elongation factor 1-gamma | - | *X. dendrorhous* | 2406 | 55.24/5.60 | 49.70/5.40 |
| 39 | cDNA5_c7663 | 51 | 4 | 40 | 60S ribosomal protein L31 | - | *X. dendrorhous* | 7112 | 20.70/10.20 | 20.30/6.40 |
| 40 | cDNA5_c1969 | 57 | 8 | 22 | Ribosomal protein S5 domain 2-like protein | - | *X. dendrorhous* | 7308 | 38.50/9.22 | 40.30/6.40 |
| 41 | cDNA6_c7649 | 61 | 7 | 12 | Aspartate-tRNA ligase | - | *X. dendrorhous* | 4614 | 68.81/5.38 | 66.90/5.70 |
| 42 | cDNA6_c6437 | 57 | 4 | 65 | SsrA-binding protein | - | *X. dendrorhous* | 4005 | 9.49/10.45 | 19.50/5.80 |
| 43 | cDNA6_c8616 | 53 | 9 | 23 | tRNA adenylyltransferase, putative | - | *X. dendrorhous* | 6412 | 49.17/9.46 | 47.00/6.20 |
|  |  |  |  |  | **Transcription and Transcription Regulation** |  |  |  |  |  |
| 44 | 74702348 | 60 | 4 | 32 | RNase H | - | *Ustilago maydis* | 2215 | 13.00/10.18 | 32.26/5.33 |
| 45 | 73918962 | 60 | 6 | 26 | Pre-mRNA-splicing factor | - | *Schizosaccharomyces pombe* | 4411 | 21.41/6.85 | 44.66/5.76 |
| 46 | 417834 | 52 | 5 | 8 | SWI/SNF complex subunit SWI3 | - | *Saccharomyces cerevisiae* | 4810 | 92.87/4.78 | 111.94/5.72 |
| 47 | 731707 | 53 | 8 | 9 | H3 lysine-4 specific | - | *Saccharomyces cerevisiae* | 6812 | 124.57/9.06 | 124.80/6.20 |
| 48 | 158564282 | 62 | 10 | 11 | Mediator of RNA polymerase II transcription sub.14 | - | *Aspergillus oryzae* | 7815 | 123.92/9.01 | 109.01/6.40 |
| 49 | 160380613 | 56 | 9 | 17 | ATP-dependent RNA helicase dbp3 | - | *Botryotinia fuckeliana* | 4606 | 65.50/8.96 | 61.28/5.64 |
| 50 | 88185298 | 77 | 8 | 18 | DNA helicase | - | *Chaetomium globosum* | 6701 | 67.64/5.88 | 76.77/6.07 |
|  |  | 65 | 7 | 17 |  |  |  | 6707 |  | 76.24/6.12 |
| 51 | 160380696 | 52 | 4 | 8 | ATP-dependent RNA helicase dbp9 | - | *Botryotinia fuckeliana* | 3613 | 67.51/9.16 | 64.04/5.50 |
| 52 | 74692018 | 55 | 7 | 11 | AGL075Cp. Regulation of transcription | - | *Ashbya gossypii* | 4608 | 67.50/8.17 | 66.05/5.75 |
| 53 | s5_c2133_p | 44 | 8 | 7 | General RNA polymerase II transcription factor | - | *X. dendrorhous* | 4812 | 133.93/5.88 | 118.50/5.60 |
| 54 | cDNA5_c7935 | 76 | 8 | 32 | Polyadenylate-binding protein | - | *X. dendrorhous* | 4117 | 37.50/5.72 | 31.80/5.70 |
| 55 | cDNA6_c7939 | 61 | 8 | 33 | Transcription factor RfeF, putative | - | *X. dendrorhous* | 6411 | 48.25/6.10 | 48.70/6.20 |
|  |  |  |  |  | **Genetic Information Processing Replication and Repair** |  |  |  |  |  |
| 56 | 189041663 | 55 | 8 | 14 | Protein ZIP2 | - | *Vanderwaltozyma polyspora* | 4708 | 85.60/7.77 | 78.68/5.65 |
| 57 | 48474713 | 50 | 7 | 13 | Meiotically up-regulated gene 72 protein | - | *Schizosaccharomyces pombe* | 4408ª | 62.80/8.92 | 43.63/5.68 |
| 58 | 74676175 | 69 | 12 | 24 | DNA polymerase alpha-associated DNA helicase A | - | *Schizosaccharomyces pombe* | 8503 | 75.35/6.74 | 52.83/7.11 |
| 59 | 74630069 | 70 | 8 | 11 | DNA replication helicase | - | *Encephalitozoon cuniculi* | 8304 | 108.05/5.87 | 41.38/7.19 |
| 60 | 74583120 | 51 | 7 | 36 | G4P04 (Fragment) | - | *Emericella nidulans* | 3228 | 24.32/8.97 | 34.64/5.44 |
| 61 | 61214661 | 50 | 3 | 20 | DNA replication complex GINS protein PSF3 | - | *Saccharomyces cerevisiae* | 1213 | 21.99/5.13 | 39.22/5.31 |
| 62 | 88181130 | 87 | 12 | 17 | Predicted protein. Exonuclease V, alpha subunit | - | *Chaetomium globosum* | 9209 | 43.71/9.62 | 35.39/9.44 |
| 63 | 58258539 | 74 | 6 | 24 | Ribonuclease H, putative | - | *Cryptococcus neoformans* | 8001 | 35.55/5.85 | 17.80/6.60 |
| 64 | s6_c1236_p | 43 | 12 | 12 | DNA topoisomerase II, putative | - | *X. dendrorhous* | 6811 | 166.80/8.09 | 118.80/6.27 |
| 65 | s6_c1477_p1 | 41 | 6 | 15 | Cell division control protein 25, putative | M | *X. dendrorhous* | 7711 | 75.20/9.43 | 72.30/6.38 |
| 66 | s6_c7188_p1 | 43 | 10 | 14 | Ku80-like protein | - | *X. dendrorhous* | 4725 | 94.60/5.10 | 105.50/5.70 |
| 67 | s5_c9012_p | 41 | 9 | 8 | DNA ATP-dependent helicase | - | *X. dendrorhous* | 5607 | 127.47/6.61 | 67.20/6.10 |
| 68 | s5_c863_p | 48 | 14 | 10 | DNA-directed RNA polymerase I | - | *X. dendrorhous* | 7810 | 182.28/6.26 | 129.50/6.30 |
| 69 | s5_c6133_p | 47 | 12 | 12 | DNA polymerase gamma, predicted | - | *X. dendrorhous* | 8506 | 61.88/9.51 | 57.70/8.20 |
| 70 | s6_c11128_p | 41 | 13 | 9 | Histone acetyltransferase, predicted | - | *X. dendrorhous* | 7515 | 164.57/6.71 | 61.00/6.40 |
| 71 | cDNA6_c7912 | 55 | 9 | 14 | Histone acetylase complex subunit | - | *X. dendrorhous* | 6728 | 84.29/9.58 | 86.00/6.30 |
| 72 | cDNA6_c3599 | 54 | 7 | 25 | DNA repair protein | - | *X. dendrorhous* | 8210 | 39.27/9.73 | 35.60/7.50 |
| 73 | xa5_c2079_p | 50 | 7 | 13 | DNA repair protein, SNF2 family | - | *X. dendrorhous* | 2716 | 86.14/8.50 | 79.30/5.40 |
| 74 | s5_c6133_p | 47 | 12 | 8 | Gamma DNA-directed DNA polymerase | - | *X. dendrorhous* | 7211 | 164.78/8.79 | 36.9/6.40 |
| 75 | cDNA5_c5428 | 50 | 4 | 51 | Putative dna polymerase protein | - | *X. dendrorhous* | 1107 | 9.86/4.87 | 29.40/5.30 |
| 76 | cDNA6_c335 | 49 | 5 | 52 | Probable ftsK/SpoIIIE family protein | M | *X. dendrorhous* | 8045 | 9.40/12.27 | 16.00/8.00 |
| 77 | cDNA5_c8002 | 52 | 4 | 29 | GTP-binding nuclear protein RAN | - | *X. dendrorhous* | 7109 | 24.41/8.99 | 31.20/6.50 |
|  |  |  |  |  | **Genetic Information Processing** |  |  |  |  |  |
| 78 | 73921582 | 51 | 5 | 11 | Calpain-like protease palB/RIM13 | - | *Aspergillus oryzae* | 4803 | 96.09/5.93 | 106.07/5.62 |
|  |  | 51 | 5 | 11 |  |  |  | 4806 |  | 105.94/5.91 |
| 79 | 187978707 | 85 | 12 | 8 | Metallopeptidase | - | *Pyrenophora tritici-repentis* | 4712 | 118.87/4.99 | 78.36/5.76 |
| 80 | 169850970 | 83 | 8 | 11 | Predicted protein. Cysteine-type endopeptidase activity | - | *Coprinopsis cinerea okayama* | 7602 | 81.31/5.46 | 63.49/6.28 |
| 81 | 74700737 | 51 | 6 | 13 | Hypothetical protein. ATP-binding, Chaperone | - | *Ustilago maydis* | 5602 | 60.13/5.71 | 61.65/5.91 |
| 82 | 74668029 | 51 | 5 | 8 | Aspartyl aminopeptidase | - | *Aspergillus fumigatus* | 5322 | 56.33/6.61 | 40.04/5.90 |
| 83 | 52000792 | 63 | 8 | 14 | T-complex protein 1 subunit delta | - | *Candida glabrata* | 4516 | 57.60/6.51 | 57.50/5.68 |
| 84 | 70987446 | 61 | 4 | 18 | Serine/threonine protein kinase (Kin28) | - | *Aspergillus fumigatus* | 7528 | 45.22/6.03 | 58.74/6.53 |
| 85 | 74589431 | 56 | 5 | 10 | Rpn6p | - | *Candida albicans* | 5601ª | 49.52/5.85 | 62.80/5.89 |
| 86 | 58262290 | 60 | 16 | 19 | Ubiquitin-protein ligase | - | *Cryptococcus neoformans* | 7510ª | 88.93/7.92 | 61.20/6.30 |
| 87 | 240275849 | 54 | 4 | 15 | Protein kinase | - | *Ajellomyces capsulatus* | 2107 | 47.44/5.98 | 31.32/5.37 |
| 88 | s6_c1168_p | 47 | 11 | 13 | Cell polarity protein, putative | M | *X. dendrorhous* | 3805 | 172.79/5.93 | 115.10/5.40 |
| 89 | 731907 | 60 | 4 | 15 | DnaJ-like protein 1 | - | *Saccharomyces cerevisiae* | 2310 | 48.65/5.12 | 42.33/5.36 |
| 90 | 145233309 | 77 | 6 | 17 | T-complex protein 1 subunit gamma | - | *Aspergillus niger* | 3229 | 59.00/5.81 | 39.27/5.46 |
| 91 | cDNA5_c7067 | 146 | 16 | 45 | HSP 60 | - | *X. dendrorhous* | 2603 | 44.15/8.410 | 47.90/7.90 |
|  |  | 102 | 9 | 23 |  | - |  | 2625 |  | 47.86/7.87 |
| 92 | cDNA6_c8249 | 56 | 6 | 10 | T-complex protein 1 | - | *X. dendrorhous* | 4604 | 73.50/9.13 | 66.20/5.60 |
| 93 | xa5_c9457_p1 | 213 | 25 | 30 | Heat shock protein, putative | - | *X. dendrorhous* | 2813 | 85.86/5.13 | 109.70/5.40 |
| 94 | cDNA5_c7020 | 103 | 16 | 27 | Hypothetical heat shock protein | - | *X. dendrorhous* | 5723 | 84.96/6.31 | 86.70/6.10 |
| 95 | xa5_c1685_p | 47 | 4 | 5 | Serine/threonine-protein kinase nrc-2 | - | *X. dendrorhous* | 7624 | 83.49/9.31 | 65.30/6.30 |
| 96 | cDNA5_c7855 | 117 | 4 | 26 | Probable mitochondrial processing peptidase beta chain precursor | - | *X. dendrorhous* | 4115 | 51.97/8.89 | 26.90/5.50 |
| 97 | cDNA5_c3491 | 49 | 4 | 21 | DnaJ homolog subfamily A member 3, mitochondrial-like | - | *X. dendrorhous* | 8208 | 30.96/9.64 | 33.90/7.20 |
| 98 | cDNA5_c7066 | 172 | 15 | 41 | Peptidase M16 | - | *X. dendrorhous* | 6401 | 46.65/7.79 | 44.50/6.20 |
| 99 | cDNA6_c9119 | 50 | 4 | 40 | HSP90 co-chaperone Cdc37 |  | *X. dendrorhous* | 9031 | 16.69/11.67 | 19.90/9.60 |
| 100 | cDNA6_c4724 | 51 | 5 | 30 | P-loop containing nucleoside triphosphate hydrolase protein |  | *X. dendrorhous* | 4112 | 14.04/10.50 | 21.40/5.60 |
| 101 | cDNA6_c6161 | 49 | 6 | 9 | Related to dual specificity protein kinase Fuz7 |  | *X. dendrorhous* | 6727 | 80.49/5.23 | 85.30/6.20 |
|  |  |  |  |  | **Metabolism: Redox** |  |  |  |  |  |
| 102 | 302416885 | 53 | 6 | 19 | Disulfide-isomerase erp38 | - | *Verticillium albo-atrum* | 7310 | 39.88/6.86 | 39.74/6.28 |
| 103 | 119413925 | 68 | 9 | 23 | Monooxygenase | - | *Neosartorya fischeri* | 4713 | 49.01/6.32 | 87.24/5.80 |
|  |  | 73 | 9 | 23 |  | - |  | 5703 |  | 87.14/5.91 |
| 104 | 121928042 | 64 | 8 | 24 | Coenzyme F420-dependent N5 | - | *Aspergillus oryzae* | 7530 | 52.08/5.58 | 51.37/6.59 |
| 105 | 121792603 | 60 | 7 | 23 | Hypothetical protein. Oxidoreductase activity | - | *Chaetomium globosum* | 4401 | 35.99/9.41 | 43.95/5.54 |
| 106 | 74680537 | 58 | 5 | 8 | Cu-oxidase | - | *Emericella nidulans* | 3606 | 76.11/5.4 | 67.83/5.39 |
| 107 | 33313426 | 135 | 11 | 41 | Alcohol dehydrogenase | - | *Phaffia rhodozyma* | 5208 | 39.06/6.07 | 39.06/6.07 |
| 108 | 121797391 | 58 | 6 | 23 | Dehydrogenases with different specificities SDR | - | *Aspergillus oryzae* | 5202 | 27.70/5.57 | 37.47/5.87 |
| 109 | 14134949 | 50 | 4 | 15 | Mn superoxide dismutase | - | *Phaffia rhodozyma* | 7108 | 22.12/6.43 | 22.12/6.43 |
| 110 | 121800584 | 52 | 5 | 21 | Electron transfer flavoprotein | - | *Aspergillus oryzae* | 8101 | 28.23/8.98 | 30.32/5.49 |
| 111 | 154313430 | 77 | 9 | 22 | Putative oxidoreductase activity | - | *Botryotinia fuckeliana* | 6515 | 46.56/6.34 | 53.45/6.25 |
| 112 | 156053626 | 65 | 5 | 25 | NADPH:quinone oxidoreductase | - | *Sclerotinia sclerotiorum* | 8310 | 36.99/8.82 | 41.36/7.66 |
| 113 | 74680498 | 68 | 6 | 18 | Cytochrome P450 protein | M | *Emericella nidulans* | 5315 | 52.86/8.61 | 39.80/6.07 |
| 114 | 261197676 | 62 | 7 | 23 | NAD binding Rossmann fold oxidoreductase | - | *Ajellomyces dermatitidis* | 3403 | 39.81/6.51 | 44.96/5.46 |
| 115 | 2499475 | 53 | 4 | 28 | Peroxiredoxin TSA2. | - | *Saccharomyces cerevisiae* | 7105 | 21.71/6.74 | 30.37/7.91 |
| 116 | 146415847 | 59 | 5 | 22 | Formate dehydrogenase NADP+ | - | *Pichia guilliermondii* | 4513 | 42.16/6.08 | 56.99/5.54 |
| 117 | 74632761 | 56 | 5 | 16 | YALI0F09097p. Potential oxidoreductase | - | *Yarrowia lipolytica* | 4310 | 38.16/5.36 | 40.06/5.54 |
| 118 | 303316063 | 56 | 4 | 16 | Hydrolase, NUDIX family protein | - | *Coccidioides posadasii* | 5205 | 40.83/5.46 | 36.93/6.12 |
| 119 | 238489279 | 59 | 5 | 19 | Monooxygenase, putative | - | *Aspergillus flavus* | 5320 | 45.84/5.93 | 40.58/6.08 |
| 120 | 70826274 | 58 | 4 | 23 | Lipoxygenase (Fragment) | - | *Aspergillus ochraceus* | 8102 | 23.97/8.28 | 29.96/6.96 |
| 121 | cDNA5_c8120 | 57 | 9 | 21 | Oxidoreductase putative | - | *X. dendrorhous* | 1424 | 47.41/5.11 | 51.40/5.30 |
| 122 | cDNA6_c6268 | 59 | 4 | 41 | Alcohol dehydrogenase fragment | - | *X. dendrorhous* | 6207 | 18.08/7.92 | 33.60/6.20 |
| 123 | cDNA6_c8412 | 56 | 6 | 19 | Protein disulfide isomerase | - | *X. dendrorhous* | 5324 | 43.58/4.64 | 41.40/6.10 |
| 124 | cDNA5_c3418 | 70 | 4 | 5 | Glutathione-disulfide reductase | - | *X. dendrorhous* | 7618 | 65.91/9.31 | 61.80/6.50 |
| 125 | cDNA5_c6845 | 51 | 7 | 15 | Probable NADPH2 dehydrogenase chain OYE2 | - | *X. dendrorhous* | 2601 | 51.68/6.12 | 64.60/5.30 |
| 126 | cDNA5_c8220 | 66 | 7 | 43 | Short-chain dehydrogenase/reductase SDR | - | *X. dendrorhous* | 2210 | 26.31/5.81 | 34.50/5.40 |
| 127 | cDNA6_c5838 | 50 | 4 | 63 | Probable thioredoxin | - | *X. dendrorhous* | 9127 | 11.60/9.62 | 19.70/9.40 |
| 128 | cDNA5_c7178 | 55 | 5 | 25 | Probable thioredoxin peroxidase | - | *X. dendrorhous* | 5313 | 30.66/7.63 | 40.90/6.10 |
| 129 | cDNA5_c7176 | 59 | 5 | 12 | Zinc-binding oxidoreductase | - | *X. dendrorhous* | 7533 | 48.44/8.28 | 51.40/6.40 |
|  |  |  |  |  | **Metabolism: Amino acid** |  |  |  |  |  |
| 130 | 74625934 | 59 | 6 | 12 | Seryl-tRNA synthetase, mitochondrial | - | *Schizosaccharomyces pombe* | 8604 | 51.69/8.68 | 59.52/7.06 |
| 131 | 2492964 | 56 | 6 | 18 | DAHP synthetase | - | *Candida albicans* | 6616 | 40.65/6.20 | 61.94/6.26 |
| 132 | 74585221 | 62 | 8 | 15 | Likely mitochondrial tyrosyl-tRNA synthetase | - | *Candida albicans* | 4512 | 56.21/6.34 | 52.14/5.75 |
| 133 | 170945133 | 65 | 11 | 12 | Carbamoylphosphate synthase large subunit | - | *Podospora anserina* | 1819 | 130.71/5.99 | 123.07/5.31 |
| 134 | 154300018 | 51 | 3 | 19 | Predicted protein Acetyltransferase | - | *Botryotinia fuckeliana* | 3112ª | 21.89/5.92 | 30.32/5.49 |
| 135 | 151942753 | 52 | 12 | 12 | Mitochondrial isoleucyl-tRNA synthetase | - | *Saccharomyces cerevisiae* | 7805 | 116.57/8.43 | 116.89/6.28 |
|  |  | 56 | 11 | 10 |  |  |  | 7808 |  | 116.65/6.30 |
|  |  | 53 | 9 | 9 |  |  |  | 7811 |  | 116.57/6.37 |
| 136 | 121928306 | 73 | 4 | 30 | RIB40 genomic DNA. Methionyl-tRNA formyltransferase | - | *Aspergillus oryzae* | 7209 | 28.83/9.59 | 36.14/6.28 |
|  |  | 52 | 4 | 30 |  | - |  | 7210 |  | 36.36/6.30 |
| 137 | 302684253 | 58 | 9 | 20 | Putative Glycine/D-amino acid oxidase | - | *Schizophyllum commune* | 4004 | 47.95/5.77 | 27.36/5.69 |
| 138 | 223635247 | 60 | 7 | 15 | Kynurenine 3-monooxygenase | M | *Pichia stipitis* | 7816 | 54.63/6.95 | 120.07/6.41 |
|  |  | 59 | 6 | 12 |  |  |  | 7817 |  | 120.06/6.43 |
|  |  | 50 | 6 | 12 |  |  |  | 7819 |  | 120.08/6.53 |
| 139 | 119413553 | 70 | 6 | 8 | Aspartyl-tRNA synthetase, cytoplasmic | - | *Neosartorya fischeri* | 6821 | 108.47/6.46 | 108.74/6.23 |
|  |  | 62 | 5 | 8 |  |  |  | 6828 |  | 108.62/6.25 |
| 140 | 302896292 | 57 | 4 | 15 | Aspartate aminotransferase putative | - | *Nectria haematococca* | 7307 | 45.31/7.72 | 40.78/6.42 |
| 141 | 30913511 | 51 | 6 | 16 | tRNA (cytosine-5-)-methyltransferase ncl1 | - | *Schizosaccharomyces pombe* | 3511ª | 45.21/6.47 | 56.97/5.48 |
| 142 | 30912640 | 60 | 5 | 13 | Acetylornithine aminotransferase, mitochondrial | - | *Schizosaccharomyces pombe* | 5410 | 48.29/8.75 | 45.45/6.09 |
| 143 | 12229964 | 57 | 7 | 12 | Phenylalanyl-tRNA ligase | - | *Schizosaccharomyces pombe* | 2517 | 67.71/5.29 | 57.05/5.36 |
| 144 | 74624697 | 50 | 2 | 66 | Histidine biosynthesis trifunctional protein | - | *Saccharomyces bayanus* | 4003 | 5.72/4.29 | 10.60/5.60 |
| 145 | 74696766 | 61 | 6 | 20 | Hypothetical protein NCU08355.1. Methyltransferase domain | - | *Neurospora crassa* | 6106 | 31.78/5.56 | 25.68/6.25 |
| 146 | 121814778 | 52 | 3 | 9 | Acetylglutamate kinase | - | *Gibberella zeae* | 3330c | 44.36/8.74 | 40.42/5.40 |
| 147 | 164423080 | 83 | 12 | 23 | NCU09184.1. Indoleamine dioxygenase | - | *Neurospora crassa* | 7510b | 74.42/6.99 | 61.20/6.30 |
| 148 | 306531020 | 57 | 6 | 15 | Arginine biosynthesis bifunctional protein | - | *Ustilago maydis* | 4607 | 53.72/6.22 | 63.42/5.69 |
| 149 | 209406130 | 98 | 10 | 34 | Glutamate dehydrogenase | - | *X. dendrorhous* | 5409 | 48.98/5.99 | 46.79/6.07 |
| 150 | 119480461 | 61 | 6 | 14 | Glutamate semialdehyde dehydrogenase | - | *Neosartorya fischeri* | 2204 | 48.89/5.37 | 37.98/5.34 |
| 151 | cDNA5_c7206 | 218 | 16 | 31 | Cobalamin-independent methionine synthase | - | *X. dendrorhous* | 4703 | 76.36/5.53 | 77.60/5.60 |
| 152 | cDNA5_c8132 | 66 | 7 | 13 | Chorismate synthase, putative | - | *X. dendrorhous* | 2606 | 46.26/7.30 | 68.50/5.30 |
| 153 | cDNA5_c7266 | 115 | 10 | 34 | Aspartate-semialdehyde dehydrogenase | - | *X. dendrorhous* | 2407 | 46.25/6.25 | 46.50/5.30 |
| 154 | cDNA6_c11418 | 132 | 15 | 24 | Arg-6 protein, partial | - | *X. dendrorhous* | 4615 | 69.46/6.94 | 66.20/5.50 |
| 155 | cDNA5_c7471 | 166 | 12 | 31 | Probable S-adenosylmethionine synthetase 2 | - | *X. dendrorhous* | 4521 | 50.971 | 58.40/5.80 |
| 156 | cDNA5_c7970 | 141 | 15 | 28 | Adenosylhomocysteinase | - | *X. dendrorhous* | 2628 | 55.29/6.11 | 66.60/5.40 |
| 157 | cDNA5_c2467 | 62 | 10 | 12 | Succinate-semialdehyde dehydrogenase mitochondrial precursor | - | *X. dendrorhous* | 7529 | 69.43/8.59 | 60.00/6.60 |
| 158 | cDNA6_c11760 | 57 | 7 | 16 | Aspartate aminotransferase | - | *X. dendrorhous* | 4612 | 48.33/8.89 | 66.90/5.80 |
| 159 | cDNA6_c8352 | 106 | 10 | 23 | Aspartate aminotransferase, mitochondrial precursor | - | *X. dendrorhous* | 2605 | 59.13/7.98 | 64.40/5.30 |
| 160 | cDNA5_c9724 | 67 | 10 | 13 | Lysyl-tRNA synthetase | - | *X. dendrorhous* | 6607 | 84.34/7.70 | 68.00/6.20 |
| 161 | cDNA6_c2201 | 168 | 16 | 36 | Glycine hydroxymethyltransferase | - | *X. dendrorhous* | 8409 | 66.59/8.21 | 48.00/7.60 |
| 162 | cDNA5_c3506 | 49 | 8 | 21 | Homocitrate synthase | - | *X. dendrorhous* | 7531 | 66.08/6.09 | 52.90/6.40 |
| 163 | cDNA6_c3151 | 99 | 10 | 23 | Argininosuccinate lyase, putative | - | *X. dendrorhous* | 4502 | 59.63/6.01 | 52.30/5.60 |
| 164 | cDNA5_c1711 | 54 | 8 | 18 | Delta-1-pyrroline-5-carboxylate dehydrogenase, mitochondrial | M | *X. dendrorhous* | 3515 | 59.49/8.49 | 52.30/5.50 |
| 165 | cDNA5_c9734 | 50 | 7 | 21 | Branched-chain-amino-acid aminotransferase 2 | - | *X. dendrorhous* | 3508 | 53.80/9.01 | 51.10/5.40 |
| 166 | cDNA5_c9075 | 112 | 11 | 19 | 2-isopropylmalate synthase | - | *X. dendrorhous* | 3723 | 74.77/5.62 | 77.20/5.40 |
| 167 | cDNA5_c9421 | 66 | 7 | 10 | Dihydroxy-acid dehydratase | - | *X. dendrorhous* | 7604 | 75.61/8.53 | 66.80/6.30 |
| 168 | cDNA5_c3415 | 52 | 6 | 19 | 3-isopropylmalate dehydrogenase | - | *X. dendrorhous* | 2505 | 52.72/5.73 | 53.30/5.30 |
|  |  |  |  |  | **Metabolism: Nucleotide** |  |  |  |  |  |
| 169 | 308191552 | 65 | 7 | 17 | Adenylosuccinate synthetase | - | *Vanderwaltozyma polyspora* | 4408b | 48.20/6.4 | 43.63/5.68 |
| 170 | 19171028 | 70 | 9 | 20 | ATP dependent DNA binding Helicase | - | *Encephalitozoon cuniculi* | 2218 | 69.02/6.07 | 37.12/5.36 |
| 171 | 121712429 | 56 | 6 | 15 | GMP synthase | - | *Aspergillus clavatus* | 3404 | 59.88/5.89 | 46.59/5.39 |
| 172 | 242214200 | 61 | 9 | 27 | Phosphoribosylaminoimidazole-succinocarboxamide synthase | - | *Postia placenta* | 5209 | 34.69/5.93 | 33.50/6.09 |
| 173 | s6_c1090_p2 | 52 | 8 | 20 | Phospho-2-dehydro-3-deoxyheptonate aldolase | - | *X. dendrorhous* | 3225 | 41.29/6.42 | 35.70/5.40 |
| 174 | 50557006 | 61 | 7 | 17 | ADK2 adenylate kinase | - | *Yarrowia lipolytica* | 7305 | 25.53/6.36 | 39.68/6.39 |
| 175 | cDNA6_c725 | 63 | 5 | 38 | SAICAR synthase-like protein | - | *X. dendrorhous* | 9126 | 14.17/10.09 | 22.00/9.60 |
| 176 | cDNA5_c3432 | 62 | 5 | 16 | Uricase (Urate oxidase) | - | *X. dendrorhous* | 5318 | 44.26/6.49 | 41.90/6.00 |
| 177 | cDNA5_c6598 | 105 | 13 | 21 | GMP synthase | - | *X. dendrorhous* | 3724 | 69.66/5.89 | 100.60/5.50 |
| 178 | cDNA5_c9922 | 44 | 7 | 12 | Bifunctional purine biosynthesis protein | - | *X. dendrorhous* | 7631 | 76.31/7.59 | 71.50/6.40 |
|  |  |  |  |  | **Metabolism: Energy Metabolism** |  |  |  |  |  |
| 179 | 74684619 | 73 | 7 | 13 | ATP synthase subunit alpha | M | *Cryptococcus neoformans* | 8507 | 58.17/9.01 | 58.20/9.01 |
| 180 | 74701945 | 156 | 18 | 46 | ATP synthase subunit beta | M | *Ustilago maydis* | 1504 | 55.69/5.28 | 55.66/5.31 |
|  |  | 132 | 15 | 43 |  |  |  | 1505 |  | 55.03/5.30 |
|  |  | 121 | 13 | 36 |  |  |  | 1506 |  | 55.33/5.29 |
|  |  | 114 | 13 | 34 |  |  |  | 1507 |  | 55.72/5.28 |
| 181 | 74634773 | 52 | 6 | 37 | NADH dehydrogenase (ubiquinone) 1 beta subcomplex 9 | M | *Yarrowia lipolytica* | 0310ª | 22.87/6.91 | 41.40/4.20 |
| 182 | BAC66640 | 74 | 10 | 11 | Vacuolar membrane ATPase subunit a precursor | M | *Saccharomyces cerevisiae* | 7812 | 113.58/6.15 | 109.14/6.38 |
| 183 | cDNA6_c8286 | 118 | 13 | 24 | V-type ATPase | M | *X. dendrorhous* | 3722 | 72.99/5.32 | 77.50/5.40 |
| 184 | cDNA6_c9791 | 56 | 5 | 51 | ATP synthase alpha chain, mitochondrial precursor | M | *X. dendrorhous* | 5108 | 9.16/6.18 | 26.00/6.10 |
| 185 | cDNA6_c9422 | 60 | 5 | 27 | Inorganic diphosphatase | - | *X. dendrorhous* | 5105 | 28.30/5.53 | 29.00/6.00 |
| 186 | cDNA5_c7092 | 85 | 8 | 14 | Vacuolar ATP synthase | M | *X. dendrorhous* | 4710 | 68.22/5.54 | 73.30/5.70 |
|  |  |  |  |  | **Metabolism: Lipid** |  |  |  |  |  |
| 187 | 121803722 | 50 | 3 | 18 | Phosphatidylinositol synthase | M | *Aspergillus oryzae* | 7111 | 30.81/8.49 | 41.40/5.49 |
| 188 | 169596590 | 67 | 8 | 20 | Fatty acid desaturase | M | *Phaeosphaeria nodorum* | 6314 | 48.84/7.79 | 39.66/6.17 |
| 189 | 14861255 | 68 | 3 | 64 | Polyketide synthase (Fragment) | - | *Polycephalomyces formosus* | 1105 | 8.61/5.15 | 29.82/5.28 |
| 190 | 46426876 | 57 | 14 | 8 | CQ798506 NID. Acetyl-CoA carboxylase, cytosolic | - | *Phaffia rhodozyma* | 2524 | 245.73/5.82 | 52.04/5.37 |
|  |  | 53 | 13 | 8 |  |  |  | 2523 |  | 52.73/5.35 |
|  |  | 65 | 14 | 8 |  |  |  | 3516 |  | 51.28/5.41 |
| 191 | 74672173 | 53 | 7 | 16 | Phosphatidylserine decarboxylase, putative | - | *Aspergillus fumigatus* | 6511 | 61.26/9.45 | 57.10/6.24 |
| 192 | 74694301 | 68 | 10 | 17 | ADR052Wp. Simil acyl-CoA synthetase | M | *Ashbya gossypii* | 4603 | 84.26/7.49 | 63.56/5.60 |
| 193 | s6_c1131_p | 83 | 7 | 23 | Acetyl-CoA C-acetyltransferase | - | *X. dendrorhous* | 6304 | 45.54/8.38 | 41.30/6.20 |
| 194 | s6_c7358_p | 47 | 11 | 8 | Fatty acid synthase | - | *X. dendrorhous* | 6604 | 223.28/6.15 | 63.60/6.20 |
|  |  |  |  |  | **Metabolism: Secondary metabolite/carotenoid biosynthesis** |  |  |  |  |  |
| 195 | 46111687 | 56 | 4 | 14 | Phytoene/squalene synthetase | - | *Gibberella zeae* | 4515 | 52.28/9.11 | 52.82/5.66 |
| 196 | 125407 | 64 | 6 | 24 | Mevalonate kinase. | - | *Saccharomyces cerevisiae* | 4609 | 48.94/5.36 | 63.47/5.77 |
| 197 | 9955387 | 63 | 6 | 17 | Squalene synthase | M | *Candida glabrata* | 5717 | 51.82/5.54 | 74.59/6.07 |
| 198 | 70993694 | 72 | 6 | 12 | Prenyltransferase | - | *Aspergillus fumigatus* | 5303 | 44.86/5.66 | 40.47/5.91 |
| 199 | 68489506 | 69 | 5 | 14 | Geranylgeranyl pyrophosphate synthase/Polyprenyl synthetase | - | *Candida albicans* | 4304 | 38.28/5.48 | 41.28/5.62 |
| 200 | 33465817 | 53 | 5 | 10 | Phytoene desaturase | - | *X. dendrorhous* | 7601 | 65.06/6.17 | 61.30/6.3 |
| 201 | 323710252 | 41 | 5 | 10 | Astaxanthin synthase | M | *X. dendrorhous* | 7501 | 62.61/6.29 | 53.3/6.30 |
| 202 | PM1619_p | 49 | 11 | 20 | Diphosphomevalonate decarboxylase | - | *X. dendrorhous* | 6308 | 43.41/575 | 41.80/6.20 |
| 203 | PM41-401_p | 43 | 4 | 9 | Phosphomevalonate kinase | - | *X. dendrorhous* | 3517 | 57.59/6.03 | 59.4/5.50 |
| 204 | x6_c2447_p1 | 51 | 6 | 18 | Cytochrome P450 reductase (crtR) gene | - | *X. dendrorhous* | 7311 | 48.29/9.92 | 41.40/6.60 |
| 205 | cDNA5_c3282 | 43 | 4 | 19 | IPP isomerase | - | *X. dendrorhous* | 6408 | 41.87/9.93 | 48.00/6.30 |
|  |  |  |  |  | **Metabolism: General** |  |  |  |  |  |
| 206 | 254580523 | 73 | 6 | 30 | Fragile histidine family. ZYRO0C13970p | - | *Zygosaccharomyces rouxii* | 4408c | 22.69/6.38 | 43.63/5.68 |
| 207 | 238054304 | 68 | 14 | 16 | Nitrite reductase [NAD(P)H] | - | *Emericella nidulans* | 3603 | 123.99/5.81 | 66.24/5.38 |
| 208 | 74695317 | 53 | 4 | 15 | ABR123Wp. Glutamine amido transferase | - | *Ashbya gossypii* | 7534 | 30.84/7.70 | 52.04/6.33 |
| 209 | 1945628 | 56 | 8 | 17 | Heat shock protein 70 | - | *Cryptococcus curvatus* | 711 | 70.22/4.98 | 74.69/4.42 |
| 210 | cDNA6_c12037 | 85 | 10 | 27 | HSP70 | - | *X. dendrorhous* | 3701 | 58.18/8.97 | 59.3/7.8 |
|  |  | 75 | 9 | 25 |  | - |  | 3715 |  | 59.3/8.3 |
|  |  | 72 | 8 | 25 |  | - |  | 3716 |  | 69.5/5.5 |
| 211 | cDNA6_c6240 | 153 | 9 | 19 | UDP-xylose synthase | - | *X. dendrorhous* | 7407 | 55.72/6.12 | 58.2/5.4 |
| 212 | 241948381 | 72 | 10 | 30 | Pyridoxin biosynthesis protein | - | *Candida dubliniensis* | 5603ª | 32.09/6.96 | 63.90/5.98 |
| 213 | 74680295 | 80 | 9 | 25 | Hypothetical protein SNZ99 | - | *Candida albicans* | 5603b | 32.15/5.76 | 63.90/5.98 |
| 214 | 70984990 | 72 | 6 | 19 | 1-aminocyclopropane-1-carboxylate deaminase | - | *Aspergillus fumigatus* | 5604 | 42.12/8.65 | 61.62/6.05 |
|  |  | 60 | 6 | 21 |  |  |  | 6608 |  | 61.49/6.18 |
| 215 | 74666885 | 55 | 4 | 9 | FMN-dependent dehydrogenase family protein | - | *Aspergillus fumigatus* | 3330d | 41.31/7.03 | 40.42/5.40 |
| 216 | 71021639 | 60 | 5 | 25 | Dephospho-CoA kinase | - | *Ustilago maydis* | 7306 | 31.64/8.67 | 39.91/6.51 |
| 217 | 39970739 | 63 | 7 | 17 | Methylcitrate dehydratase | - | *Magnaporthe oryzae* | 3607 | 62.56/7.94 | 64.40/5.42 |
| 218 | s6_c1356_p | 41 | 9 | 11 | Heat shock protein, putative | - | *X. dendrorhous* | 7801 | 108.35/6.48 | 115.00/6.30 |
| 219 | 225563143 | 67 | 6 | 15 | Arginine N-methyltransferase | - | *Ajellomyces capsulata* | 2611 | 47.94/4.54 | 68.35/5.36 |
| 220 | cDNA5_c7828 | 89 | 16 | 17 | Probable myo-inositol 1-phosphate synthase | - | *X. dendrorhous* | 6832 | 120.86/9.91 | 111.80/6.30 |
| 221 | cDNA6_c11985 | 50 | 7 | 21 | NAD(P)-binding protein | - | *X. dendrorhous* | 5408 | 96.87/10.11 | 45.00/6.01 |
| 222 | cDNA5_c7159 | 69 | 10 | 16 | Fumarate reductase | - | *X. dendrorhous* | 6729 | 68.27/6.33 | 74.20/6.20 |
| 223 | cDNA5_c7431 | 68 | 7 | 17 | Putative heat shock protein 70 | - | *X. dendrorhous* | 7722 | 31.66/7.18 | 104.40/6.40 |
| 224 | cDNA6_c7862 | 58 | 6 | 20 | Phospholipid-translocating ATPase | M | *X. dendrorhous* | 5616 | 53.56/9.57 | 65.40/5.90 |
| 225 | cDNA5_c7162 | 114 | 12 | 29 | Ketol-acid reductoisomerase | - | *X. dendrorhous* | 8516 | 58.07/9.11 | 57.30/7.50 |
| 226 | cDNA5_c9973 | 48 | 4 | 14 | Glutathione S-transferase Gst3 | - | *X. dendrorhous* | 8504 | 48.76/10.70 | 52.90/7.30 |
| 227 | cDNA5_c3757 | 51 | 4 | 21 | Putative ubiquinone biosynthesis monooxygenase Coq6 | - | *X. dendrorhous* | 6101 | 21.29/6.40 | 29.00/6.10 |
| 228 | cDNA6_c8354 | 64 | 6 | 20 | Phosphoric monoester hydrolase | - | *X. dendrorhous* | 7404 | 40.85/11.43 | 43.90/6.40 |
| 229 | cDNA5_c7493 | 56 | 7 | 15 | Putative heat shock protein STI1 | - | *X. dendrorhous* | 4514 | 59.56/5.91 | 53.00/5.50 |
|  |  |  |  |  | **Metabolism: Carbohydrate** |  |  |  |  |  |
| 230 | 116199163 | 74 | 10 | 26 | Acetyl-CoA synthetase | - | *Chaetomium globosum* | 2319 | 60.08/6.33 | 39.71/5.37 |
| 231 | 74702395 | 57 | 6 | 7 | Alpha-glucosidases | - | *Ustilago maydis* | 2614 | 116.71/5.68 | 66.94/5.36 |
|  |  | 54 | 5 | 5 |  |  |  | 2619 |  | 67.14/5.33 |
|  |  | 53 | 5 | 4 |  |  |  | 2621 |  | 66.82/5.35 |
|  |  | 49 | 4 | 3 |  |  |  | 2622 |  | 67.05/5.34 |
|  |  | 47 | 3 | 3 |  |  |  | 2623 |  | 67.16/5.34 |
| 232 | 119409891 | 60 | 7 | 15 | Melibiase, putative | - | *Neosartorya fischeri* | 7625 | 80.84/5.84 | 63.12/6.39 |
| 233 | 169853382 | 59 | 8 | 17 | Citrate synthase | - | *Coprinopsis cinerea* | 6503 | 51.79/7.70 | 56.86/6.17 |
| 234 | 464368 | 53 | 7 | 14 | Phosphoglucomutase-1 | - | *Saccharomyces cerevisiae* | 7519 | 63.47/6.82 | 56.03/6.39 |
| 235 | 74692484 | 75 | 9 | 12 | Phosphorylase | - | *Ashbya gossypii* | 4602 | 102.84/5.44 | 62.63/6.08 |
| 236 | 74606559 | 58 | 6 | 18 | KLLA0D04510p.Carbohydrate_kinase_pred_CS | - | *Kluyveromyces lactis* | 5517 | 36.81/5.80 | 56.28/5.84 |
| 237 | 74637885 | 66 | 6 | 12 | Neutral trehalase | - | *Candida glabrata* | 2309 | 86.80/6.54 | 40.53/5.36 |
| 238 | 74705139 | 65 | 6 | 12 | Glucokinase | - | *Trichoderma reesei* | 6609 | 59.94/5.26 | 62.39/6.21 |
| 239 | 121807791 | 55 | 3 | 13 | Phosphoglycerate kinase | - | *Rhizopus oryzae* | 4201 | 44.75/6.29 | 34.09/5.53 |
| 240 | 3122121 | 90 | 11 | 35 | GAPDH | - | *Phaffia rhodozyma* | 4312 | 36.33/5.79 | 40.10/5.79 |
|  |  | 79 | 8 | 25 |  | 0 |  | 5314 |  | 40.00/6.10 |
| 241 | 74670647 | 64 | 6 | 14 | ATP-citrate lyase subunit (Acl) | - | *Aspergillus fumigatus* | 4413 | 52.99/5.88 | 48.76/5.77 |
| 242 | 30912748 | 65 | 6 | 14 | ATP-citrate synthase subunit 1 | - | *Schizosaccharomyces pombe* | 4104 | 67.84/8.11 | 29.12/5.58 |
| 243 | 302689959 | 76 | 8 | 21 | Transaldolase | - | *Schizophyllum commune* | 2213 | 35.99/5.23 | 39.33/5.37 |
| 244 | cDNA5_c7133 | 110 | 11 | 20 | Isocitrate lyase | - | *X. dendrorhous* | 8202 | 66.28/8.73 | 39.54/6.67 |
| 245 | 121808353 | 51 | 5 | 12 | Beta-fructosidase (Fragment) | - | *Saccharomyces cariocanus* | 5601b | 58.64/4.56 | 62.80/5.89 |
| 246 | cDNA6_c8483 | 86 | 10 | 17 | Succinate dehydrogenase (ubiquinone) | M | *X. dendrorhous* | 6610 | 70.94/6.46 | 67.09/6.21 |
| 247 | 70991192 | 57 | 5 | 22 | Pyruvate dehydrogenase | - | *Aspergillus fumigatus* | 6403 | 41.68/6.36 | 45.34/6.24 |
| 248 | 242794316 | 62 | 8 | 19 | Succinyl-CoA synthetase beta subunit | - | *Talaromyces stipitatus* | 5509 | 48.59/5.70 | 57.09/6.06 |
| 249 | 156836648 | 55 | 5 | 12 | Fructose-bisphosphate aldolase putative | - | *Vanderwaltozyma polyspora* | 3511b | 39.45/5.69 | 56.97/5.48 |
| 250 | s5_c1234_p2 | 41 | 5 | 12 | NAD-dependent formate dehydrogenase | - | *X. dendrorhous* | 7312 | 40.22/6.38 | 39.80/6.40 |
| 251 | s6_c1550_p2 | 112 | 11 | 45 | NADP+ malate dehydrogenase | - | *X. dendrorhous* | 3206 | 34.83./5.18 | 36.10/5.4 |
| 252 | s6_c11170_p | 56 | 8 | 7 | Pyruvate carboxylase | - | *X. dendrorhous* | 6809 | 130.84/5.93 | 124.80/6.20 |
| 253 | 254568772 | 54 | 5 | 10 | Isocitrate dehydrogenase [NADP] | - | *Pichia pastoris* | 6611 | 48.83/5.56 | 64.77/6.25 |
| 254 | s6_c1090_p1 | 42 | 4 | 10 | Oxoglutarate dehydrogenase, predicted | - | *X. dendrorhous* | 7532 | 56.40/6.11 | 52.50/6.30 |
| 255 | 156848406 | 73 | 9 | 19 | Enolase | - | *Vanderwaltozyma polyspora* | 3331 | 46.87/5.55 | 40.97/5.49 |
| 256 | 170087368 | 54 | 5 | 14 | Arylformamidase | - | *Laccaria bicolor* | 6210 | 47.27/6.50 | 39.27/6.23 |
| 257 | 255731576 | 58 | 4 | 14 | Aldehyde dehydrogenase | - | *Candida tropicalis* | 7629 | 74.48/6.78 | 63.12/6.36 |
| 258 | cDNA6_c1144 | 124 | 15 | 23 | Aconitase | - | *X. dendrorhous* | 7725 | 94.56/6.37 | 82.80/6.30 |
| 259 | cDNA5_c3341 | 73 | 10 | 18 | Glucose-6-phosphate isomerase | - | *X. dendrorhous* | 4519 | 69.01/5.95 | 59.80/5.60 |
| 260 | qi_344324259 | 170 | 4 | 13 | Pyruvate decarboxylase | - | *X. dendrorhous* | 4605 | 66.23/5.21 | 68.00/5.60 |
| 261 | cDNA6_c12055 | 139 | 17 | 34 | Transketolase | - | *X. dendrorhous* | 5701 | 82.31/5.95 | 73.20/5.80 |
| 262 | cDNA5_c7706 | 129 | 14 | 38 | 6-phosphogluconate dehydrogenase | - | *X. dendrorhous* | 604 | 54.70/5.67 | 62.80/4.60 |
| 263 | cDNA6_c5077 | 181 | 18 | 37 | Aldehyde dehydrogenase [NAD(P)+] | - | *X. dendrorhous* | 4717 | 69.43/6.90 | 70.90/5.80 |
| 264 | cDNA5_c2197 | 107 | 7 | 13 | Pyruvate kinase | - | *X. dendrorhous* | 5605 | 69.76/8.50 | 62.60/6.10 |
| 265 | cDNA5_c9792 | 114 | 13 | 22 | Glucose-6-P dehydrogenase | - | *X. dendrorhous* | 5617 | 70.83/6.14 | 62.50/60 |
| 266 | cDNA5_c9917 | 90 | 10 | 18 | Dihydrolipoamide dehydrogenase | - | *X. dendrorhous* | 7728 | 80.24/9.18 | 85.00/6.40 |
| 267 | cDNA6_c9017 | 58 | 6 | 30 | UDP-glucose/GDP-mannose dehydrogenase | - | *X. dendrorhous* | 8113 | 28.38/6.19 | 30.80/6.80 |
| 268 | cDNA5_c7877 | 140 | 8 | 41 | Malate dehydrogenase | - | *X. dendrorhous* | 7206 | 35.35/6.17 | 38.40/6.40 |
| 269 | cDNA6_c9294 | 87 | 8 | 34 | Fructose-1,6-bisphosphatase | - | *X. dendrorhous* | 7204 | 33.81/5.50 | 39.60/6.40 |
| 270 | cDNA5_c7940 | 91 | 7 | 26 | Triose phosphate isomerase | - | *X. dendrorhous* | 3209 | 36.08/6.77 | 34.90/5.50 |
| 271 | xa5_c9071_p1 | 58 | 8 | 16 | Acetate-CoA ligase ADP putative | - | *X. dendrorhous* | 7719 | 73.83/6.23 | 68.80/6.30 |
| 272 | xa5_c5628_p | 55 | 6 | 6 | 2-oxoglutarate dehydrogenase complex E1, mitochondrial precursor | - | *X. dendrorhous* | 7829 | 113.84/6.28 | 111.70/6.30 |
| 273 | cDNA5_c6982 | 83 | 7 | 25 | Putative isocitrate dehydrogenase NAD+ | - | *X. dendrorhous* | 7301 | 47.75/9.23 | 41.80/6.30 |
| 274 | cDNA6_c7605 | 155 | 18 | 38 | Phosphoenolpyruvate carboxykinase | - | *X. dendrorhous* | 4613 | 69.50/6.50 | 65.60/5.70 |
| 275 | cDNA6_c5022 | 100 | 11 | 14 | Related to NADP-dependent malic enzyme | - | *X. dendrorhous* | 4706 | 80.73/9.30 | 74.60/5.60 |
| 276 | cDNA6_c9127 | 155 | 14 | 32 | Beta-phosphoglucomutase | - | *X. dendrorhous* | 3601 | 53.86/4.97 | 64.60/5.40 |
| 277 | cDNA6_c9105 | 90 | 9 | 27 | Dihydrolipoyllysine-residue acetyltransferase | - | *X. dendrorhous* | 2620 | 51.18/8.68 | 62.90/5.40 |
| 278 | xa5_c9262_p | 246 | 25 | 47 | Acyl-CoA carboxylate CoA-transferase | - | *X. dendrorhous* | 6516 | 58.38/6.15 | 59.20/6.20 |
| 279 | cDNA5_c10331 | 54 | 6 | 14 | Eukaryotic phosphomannomutase | - | *X. dendrorhous* | 2308 | 45.69/6.84 | 40.00/5.40 |
| 280 | cDNA5_c800 | 53 | 3 | 43 | Endo-1,3(4)-beta-glucanase | - | *Phaffia rhodozyma* | 6109 | 7.29/6.73 | 20.20/6.30 |
| 281 | cDNA5_c10048 | 91 | 10 | 22 | Phosphoglycerate mutase | - | *X. dendrorhous* | 4520 | 67.85/6.34 | 59.80/5.60 |
| 282 | cDNA5_c8065 | 112 | 10 | 50 | 6-phosphogluconolactonase | - | *X. dendrorhous* | 3218 | 35.40/5.17 | 34.80/5.40 |
| 283 | cDNA6_c7055 | 50 | 3 | 40 | Ribose-5-phosphate isomerase | - | *X. dendrorhous* | 7110 | 8.97/9.51 | 30.00/6.50 |
| 284 | cDNA5_c7218 | 133 | 14 | 39 | Fumarate hydratase | - | *X. dendrorhous* | 1610 | 53.72/5.65 | 6.70/5.30 |
| 285 | cDNA5_c10078 | 57 | 7 | 23 | Dihydrolipoamide succinyltransferase, putative | - | *X. dendrorhous* | 6318 | 38.51/5.81 | 41.90/6.20 |
| 286 | cDNA5_c7144 | 77 | 8 | 21 | Mitochondrial pyruvate dehydrogenase E1 component beta subunit | - | *X. dendrorhous* | 2624 | 45.76/5.29 | 62.00/5.30 |
| 287 | cDNA5_c8099 | 58 | 4 | 19 | Hexokinase | - | *X. dendrorhous* | 5206 | 28.46/6.42 | 32.60/6.60 |
| 288 | cDNA6_c8159 | 65 | 7 | 19 | UDP-glucose epimerase | - | *X. dendrorhous* | 3618 | 53.13/6.14 | 67.60/5.40 |
| 289 | cDNA5_c7302 | 58 | 9 | 22 | Pyruvate dehydrogenase e1 component α subunit | - | *X. dendrorhous* | 2514 | 46.13/7.66 | 59.90/5.40 |
|  |  |  |  |  | **Unknown** |  |  |  |  |  |
| 290 | 74669512 | 65 | 9 | 14 | Conserved hypothetical protein | - | *Aspergillus fumigatus* | 2709 | 90.26/9.33 | 73.18/5.33 |
|  |  | 61 | 8 | 12 |  |  |  | 2710 |  | 74.25/5.33 |
|  |  | 54 | 6 | 9 |  |  |  | 2711 |  | 73.10/5.34 |
| 291 | 150866465 | 87 | 14 | 23 | PICST_33329 | - | *Pichia stipitis* | 8714 | 61.40/5.66 | 75.87/7.39 |
| 292 | 74624477 | 72 | 5 | 18 | UPF0553 protein C589.05c. | - | *Schizosaccharomyces pombe* | 7715 | 40.33/6.34 | 71.42/6.40 |
| 293 | 121778823 | 70 | 9 | 21 | Hypothetical protein | - | *Chaetomium globosum* | 6603b | 48.78/9.74 | 67.15/6.15 |
| 294 | 121779248 | 68 | 10 | 18 | Hypothetical protein | - | *Chaetomium globosum* | 6601 | 69.86/7.23 | 65.31/6.12 |
| 295 | 299790662 | 68 | 7 | 8 | AFL122Wp | - | *Ashbya gossypii* | 4422 | 57.74/10.06 | 47.08/5.71 |
|  |  | 68 | 7 | 8 |  |  |  | 5416ª |  | 46.90/5.98 |
| 296 | 303321790 | 70 | 8 | 16 | CPC735_040080 | - | *Coccidioides posadasii* | 5316 | 44.93/6.84 | 40.00/6.00 |
| 297 | 74676312 | 62 | 5 | 40 | Putative Kinase | - | *Saccharomyces cerevisiae* | 6102 | 27.45/6.45 | 28.17/6.16 |
| 298 | 74634458 | 50 | 6 | 36 | YALI0D17292p | - | *Yarrowia lipolytica* | 6110 | 16.90/7.00 | 22.36/6.16 |
| 299 | 74624918 | 67 | 7 | 26 | B24M22.220 | - | *Neurospora crassa* | 1316 | 46.95/5.64 | 40.18/5.32 |
| 300 | 121800089 | 52 | 4 | 17 | Predicted protein | - | *Aspergillus oryzae* | 3503 | 43.71/4.46 | 60.54/5.40 |
| 301 | 146323476 | 66 | 4 | 23 | Conserved hypothetical protein | - | *Aspergillus fumigatus* | 8205 | 38.46/9.08 | 30.37/7.91 |
| 302 | 85083936 | 54 | 5 | 5 | NCU00091 | - | *Neurospora crassa* | 2821 | 122.60/6.69 | 122.18/5.34 |
| 303 | 261195963 | 64 | 6 | 30 | Conserved hypothetical protein | - | *Ajellomyces dermatitidis* | 5304 | 32.94/7.08 | 41.42/5.94 |
| 304 | 12043549 | 72 | 5 | 18 | SPAC589.05c | - | *Schizosaccharomyces pombe* | 8501 | 40.33/6.34 | 52.78/6.63 |
| 305 | 50554837 | 56 | 5 | 16 | YALI0F00616p | - | *Yarrowia lipolytica* | 3615 | 41.13/5.92 | 61.38/5.49 |
| 306 | 115388093 | 71 | 10 | 22 | Hypothetical protein | - | *Aspergillus terreus* | 2404 | 39.70/4.54 | 44.46/5.34 |
| 307 | 242820978 | 70 | 6 | 15 | Hypothetical protein TSTA_000120 | - | *Talaromyces stipitatus* | 2321b | 47.77/5.07 | 40.04/5.35 |
| 308 | 303322178 | 71 | 7 | 33 | Heat repeat containing protein | - | *Coccidioides posadasii* | 3112b | 23.70/4.61 | 30.32/5.49 |
| 309 | 119484962 | 67 | 6 | 12 | C6 zinc finger domain protein | - | *Neosartorya fischeri* | 5416b | 50.36/8.95 | 46.90/5.98 |
| 310 | 169766802 | 54 | 5 | 35 | Predicted protein | - | *Aspergillus oryzae* | 0310b | 21.41/5.23 | 41.40/4.20 |
| 311 | s6_c1795_p1 | 62 | 12 | 18 | Hypothetical protein | - | *X. dendrorhous* | 7508 | 56.60/6.30 | 65.45/5.09 |
| 312 | cDNA6_c10494 | 57 | 4 | 54 | Hypothetical protein | - | *X. dendrorhous* | 1001 | 10.63/4.36 | 18.10/5.30 |
| 313 | cDNA5_c5906 | 51 | 4 | 54 | Hypothetical protein | - | *X. dendrorhous* | 7006 | 10.84/9.68 | 11.20/6.30 |
| 314 | x6_c11342_p | 49 | 8 | 10 | Predicted protein | - | *X. dendrorhous* | 8712 | 101.00/9.93 | 98.90/8.30 |
| 315 | cDNA6_c13352 | 50 | 4 | 51 | Hypothetical protein | - | *X. dendrorhous* | 3002 | 7.31/11.74 | 17.80/5.40 |
| 316 | cDNA6_c4369 | 68 | 8 | 28 | Hypothetical protein | - | *X. dendrorhous* | 3230 | 34.04/8.13 | 32.20/5.40 |
| 317 | cDNA6_c7911 | 148 | 20 | 27 | Hypothetical protein | - | *X. dendrorhous* | 4724 | 97.05/6.05 | 105.40/5.60 |
| 318 | x6_c3491_p | 85 | 10 | 20 | Hypothetical protein | - | *X. dendrorhous* | 7723 | 85.31/5.07 | 104.60/6.40 |
| 319 | cDNA5_c8273 | 49 | 4 | 52 | Hypothetical protein | - | *X. dendrorhous* | 2005 | 12.71/5.36 | 10.60/5.40 |
| 320 | cDNA5_c3491 | 49 | 4 | 21 | Hypothetical protein | - | *X. dendrorhous* | 8116 | 30.96/9.64 | 30.80/7.30 |
| 321 | cDNA6_c7998 | 55 | 9 | 30 | MT-A70-domain-containing protein | - | *X. dendrorhous* | 6103 | 31.22/9.37 | 26.50/6.20 |
| 322 | cDNA6_c5197 | 51 | 4 | 26 | Hypothetical protein | - | *X. dendrorhous* | 6111 | 19.19/9.69 | 23.80/6.20 |
| 323 | cDNA5_c3909 | 48 | 4 | 30 | Hypothetical protein | - | *X. dendrorhous* | 4113 | 23.60/9.93 | 23.60/5.60 |
| 324 | x6_c11675_p | 49 | 5 | 11 | Uncharacterized protein | - | *X. dendrorhous* | 7705 | 68.27/8.43 | 72.50/6.30 |
| 325 | cDNA6_c7197 | 60 | 12 | 9 | Hypothetical protein | - | *X. dendrorhous* | 7830 | 191.46/11.7 | 111.3/6.30 |
| 326 | cDNA5_c7952 | 54 | 5 | 34 | Hypothetical protein | - | *X. dendrorhous* | 5107 | 19.90/6.11 | 22.70/5.90 |
| 327 | cDNA6_c4369 | 68 | 8 | 28 | Hypothetical protein | - | *X. dendrorhous* | 3111 | 34.04/8.13 | 29.20/5.40 |
| 328 | cDNA5_c10414 | 110 | 10 | 48 | Hypothetical protein | - | *X. dendrorhous* | 8209 | 26.13/5.82 | 32.50/7.00 |
| 329 | cDNA6_c2511 | 51 | 9 | 15 | Hypothetical protein | - | *X. dendrorhous* | 5618 | 64.36/10.71 | 65.40/6.00 |

All identified non-redundant proteins were manually assigned N°s. The IDs correspond to the accession numbers from the NCBI database and contigs of *X. dendrorhous*. The score, peptide and coverage values correspond to the data from the MASCOT software analysis. Assignments were made according to Swiss-Prot and KEGG. CC corresponds to cellular component localization. The spot N° data were generated by PDQuest software. Theoretical Mr/pI data were obtained from the MASCOT protein identification, and experimental Mr/pI data were calculated by PDQuest. Mr, molecular mass; pI, isoelectric point. Capital letters in spot N°s; indicate overlapping spots.
