# Supplementary material for: Proteomic and metabolomic analysis of the carotenogenic yeast Xanthophyllomyces dendrorhous using different carbon sources
Source: BMC Genomics. 2015 Apr 12;16(1):289. doi: 10.1186/s12864-015-1484-6 (PMC4404605; doi:10.1186/s12864-015-1484-6)
Supplement: Additional file 3: Table S2. — X. dendrorhous proteins differential abundance when cultured in glucose or succinate. Differentially regulated proteins observed during X. dendrorhous growth are shown. [file 12864_2015_1484_MOESM3_ESM.docx]

**Table S2. X. dendrorhous proteins differential abundance when cultured in glucose or succinate.**

| **A) Differentially regulated proteins in MM glucose** | | | | |
| --- | --- | --- | --- | --- |
|  |  | ^c^**Fold Change** | | |
| ^a^**SSP** | ^b^**Assignment** | **E.E/L** | **E.L/L** | **S/L** |
|  |  |  |  |  |
|  | **Cellular Processes: Transport and Motor Proteins** |  |  |  |
| 6818 | Putative coatomer subunit alpha | 7.41 | 5.65 | **8.30** |
| 8711 | Myosin -associated protein | 5.56 | 5.05 | **3.95** |
| 5728 | Golgi transport protein | 1.02 | **-3.12** | -1.24 |
| 2712 | SS1G_01912 | -1.81 | -3.43 | -2.45 |
|  | **Environmental Information Processing: Signal Transduction** | | |  |
| 3815 | Serine/threonine-protein phosphatase PP1-1 | -4.39 | -5.54 | 1.66 |
| 0127 | 14-3-3. DNA damage checkpoint protein | -3.16 | -1.11 | **12.09** |
| 0128 | 14-3-3. DNA damage checkpoint protein | 1.00 | 2.98 | 10.63 |
|  | **Genetic Information Processing** |  |  |  |
| 9206 | Ribosomal_L15 | -2.40 | -3.14 | -1.94 |
| 7815 | Mediator of RNA polymerase II transcription sub.14 | 2.13 | 1.73 | **2.64** |
| 7711 | Cell division control protein 25, putative | 2.98 | 2.30 | 2.97 |
| 7515 | Histone acetyltransferase, predicted | 1.43 | 3.47 | **2.98** |
| 7112 | 60S ribosomal protein L31 | **-5.26** | -7.45 | -1.06 |
| 4005 | SsrA-binding protein | **-13.25** | -2.48 | -1.93 |
| 2603 | HSP 60 | 2.41 | 4.78 | **3.27** |
| 5723 | Hypothetical heat shock protein | -1.61 | -2.14 | -2.79 |
|  | **Metabolism** |  |  |  |
| 7407 | UDP-xylose synthase | 1.20 | 1.11 | **2.16** |
| 4710 | ATP synthase subunit beta | -1.70 | 1.82 | **-2.65** |
|  | **Metabolism: Lipid and Carbohydrate** | |  |  |
| 3516 | CQ798506 NID. Acetyl-CoA carboxylase, cytosolic | -1.33 | **-2.96** | -1.42 |
| 7519 | Phosphoglucomutase-1 | 1.20 | **3.23** | 1.30 |
| 6109 | Endo-1,3(4)-beta-glucanase | **-4.18** | **-21.30** | -1.32 |
| 7110 | Ribose-5-phosphate isomerase | 4.27 | -2.69 | **4.46** |
| 1610 | Fumarate hydratase | **2.04** | 1.16 | 4.57 |
|  | **Metabolism: Secondary metabolite/carotenoid biosynthesis** | | |  |
| 4515 | Phytoene/squalene synthetase | 1.48 | **2.50** | 1.44 |
| 6308 | Diphosphomevalonate decarboxylase | -1.16 | 2.16 | 2.61 |
|  | **Metabolism: Redox** |  |  |  |
| 5703 | Monooxygenase | -1.60 | 1.40 | **1.76** |
| 4401 | Oxidoreductase | 1.80 | **-6.10** | **-4.40** |
| 5202 | Dehydrogenases with different specificities SDR | -1.26 | -7.59 | 1.36 |
| 7108 | Sequence 6 from Patent EP1111067. Mn superoxide dismutase | -2.54 | **1.52** | 1.09 |
| 5315 | Cytochrome P450 protein | -1.14 | **1.50** | -1.64 |
| 2601 | Probable NADPH2 dehydrogenase chain OYE2 | **3.41** | 1.47 | 3.45 |
|  | **Metabolism: Amino acid** |  |  |  |
| 8604 | Seryl-tRNA synthetase, mitochondrial | 3.18 | 3.21 | **4.93** |
| 7209 | RIB40 genomic DNA. Methionyl-tRNA formyltransferase | 24.73 | 31.44 | 19.27 |
| 7816 | Kynurenine 3-monooxygenase | 5.10 | 6.55 | 7.32 |
| 7817 | Kynurenine 3-monooxygenase | 6.64 | 10.01 | **12.01** |
| 5409 | Glutamate dehydrogenase | 1.73 | 1.25 | 3.14 |
| 2605 | Aspartate aminotransferase, mitochondrial precursor | -1.01 | -7.30 | **-1.15** |
| 4502 | Argininosuccinate lyase, putative | 1.39 | -2.31 | -2.66 |
|  | **Unknown** |  |  |  |
| 6111 | Hypothetical protein | **-5.15** | -13.28 | -2.64 |
| 5107 | Hypothetical protein | **-10.97** | -7.57 | -3.78 |

^a^ SSP numbers were assigned by PDQuest software analysis. ^b^ Identifications were obtained using the Swiss-Prot and KEGG Pathways databases and contigs of *X. dendrorhous* genomic DNA. ^c^ Mean fold changes compared with the 24 h cultures. Statistical significance was estimated by *t*-test (p <0.02) which is shown as underlined values and the Benjamini-Hochberg (p <0.05) correction shown as bold values. L: lag phase, EE: Early exponential, EL: Late exponential, S: Stationary.

| **B) Differentially regulated proteins in MM succinate** | | | | |
| --- | --- | --- | --- | --- |
|  |  | ^c^**Fold Change** | | |
| ^a^**SSP** | ^b^**Assignment** | **E.E/L** | **E.L/L** | **S/L** |
|  |  |  |  |  |
|  | **Cellular Processes: Transport and Motor Proteins** |  |  |  |
| 6813 | Putative coatomer subunit alpha | 13.41 | **4.45** | **5.76** |
| 7408 | KIP1 kinesin- related protein | -4.05 | -2.01 | -2.90 |
| 5201 | Fimbrin | **-3.02** | -1.59 | -1.28 |
|  | **Environmental Information Processing** | | |  |
| 5515 | Negative regulator of the PHO system. Ser-thr kinase | 3.29 | 3.60 | 2.64 |
| 5417 | Eukaryotic translation initiation factor 3 subunit H | **24.79** | 77.54 | 10.13 |
| 6707 | DNA helicase | 7.66 | 2.27 | **2.84** |
| 3613 | ATP-dependent RNA helicase dbp9 | 15.33 | **6.55** | 5.12 |
| 2107 | Protein kinase | -5.12 | **-6.41** | -3.34 |
| 2310 | DnaJ-like protein 1 | 1.27 | **-5.75** | -1.23 |
| 3229 | T-complex protein 1 subunit gamma | -3.30 | -2.88 | **-2.30** |
|  | **Metabolism** |  |  |  |
| 3716 | HSP70 | 1.08 | -1.52 | 1.22 |
| 2611 | Arginine N-methyltransferase | **-3.11** | -2.40 | **-2.62** |
| 4710 | Vacuolar ATP synthase | -1.46 | -1.92 | 1.02 |
|  | **Metabolism: Carbohydrate** |  |  |  |
| 6503 | Citrate synthase | **4.80** | 2.80 | 2.29 |
| 7519 | Phosphoglucomutase-1 | 2.02 | -1.05 | **2.45** |
| 2309 | Neutral trehalase | -1.85 | -1.21 | 4.66 |
| 4312 | GAPDH | 3.25 | 5.26 | 2.45 |
| 5314 | GAPDH | 9.37 | **10.65** | 2.92 |
| 8202 | Isocitrate lyase | -4.00 | **-2.15** | -2.87 |
| 6610 | Succinate dehydrogenase (ubiquinone) | **4.49** | 5.12 | 6.39 |
| 6403 | Pyruvate dehydrogenase | 12.23 | 35.34 | **11.61** |
| 5509 | Succinyl-CoA synthetase beta subunit | **11.49** | 11.37 | 10.06 |
| 3331 | Enolase | **-2.97** | -1.98 | -2.62 |
| 4520 | Phosphoglycerate mutase | 1.27 | 2.59 | **9.49** |
|  | **Metabolism: Secondary metabolite/carotenoid biosynthesis** | | |  |
| 4609 | Mevalonate kinase. | 2.53 | **3.59** | 2.23 |
| 5717 | Squalene synthase | 2.67 | **2.23** | 2.18 |
| 5303 | Prenyltransferase | **-3.13** | -5.88 | -1.38 |
| 4304 | Geranylgeranyl pyrophosphate synthase/Polyprenyl synthetase | 3.55 | 2.10 | **4.57** |
| 7601 | Phytoene desaturase | -1.25 | -5.48 | 3.55 |
| 7501 | Astaxanthin synthase | **3.68** | 1.55 | 3.12 |
|  | **Metabolism: Redox** |  |  |  |
| 4713 | Monooxygenase | 1.11 | **1.43** | 1.36 |
| 4401 | Oxidoreductase | **-1.57** | -3.00 | -1.37 |
| 5202 | Dehydrogenases with different specificities SDR | **-2.75** | **-16.08** | -2.11 |
| 8310 | NADPH:quinone oxidoreductase | -2.39 | -1.48 | 1.08 |
| 5315 | Cytochrome P450 protein | **2.54** | 1.41 | 1.40 |
| 5320 | Monooxygenase, putative | -2.18 | -1.36 | -1.28 |
| 9127 | Probable thioredoxin | **20.65** | -1.47 | 3.28 |
|  | **Metabolism:Amino acid and nucleotides** |  |  |  |
| 8604 | Seryl-tRNA synthetase, mitochondrial | **-5.80** | -1.89 | -3.72 |
| 1819 | Carbamoylphosphate synthase large subunit | -1.69 | -2.14 | **-4.17** |
| 7210 | RIB40 genomic DNA. Methionyl-tRNA formyltransferase | **-4.26** | -1.61 | -2.39 |
| 5410 | Acetylornithine aminotransferase, mitochondrial | **2.31** | 1.89 | 1.82 |
| 5409 | Glutamate dehydrogenase | 2.50 | 3.86 | **1.72** |
| 3225 | Phospho-2-dehydro-3-deoxyheptonate aldolase | -2.41 | -3.67 | -3.53 |
| 7305 | ADK2 adenylate kinase | -2.66 | **-6.43** | -1.98 |
| 2605 | Aspartate aminotransferase, mitochondrial precursor | -1.93 | -1.88 | **-2.15** |
|  | **Unknown** |  |  |  |
| 0310 | Predicted protein | **2.06** | **8.03** | 1.16 |
| 3002 | Hypothetical protein | 1.88 | 1.87 | **6.50** |

^a^ SSP numbers were assigned by PDQuest software analysis. ^b^ Identifications were obtained using the Swiss-Prot and KEGG Pathways databases and contigs of *X. dendrorhous* genomic DNA. ^c^ Mean fold changes compared with the 24 h cultures. Statistical significance was estimated by *t*-test (p <0.02) which is shown as underlined values and the Benjamini-Hochberg (p <0.05) correction shown as bold values. L: lag phase, EE: Early exponential, EL: Late exponential, S: Stationary.
