# Supplementary material for: Proteomic and metabolomic analysis of the carotenogenic yeast Xanthophyllomyces dendrorhous using different carbon sources
Source: BMC Genomics. 2015 Apr 12;16(1):289. doi: 10.1186/s12864-015-1484-6 (PMC4404605; doi:10.1186/s12864-015-1484-6)
Supplement: Additional file 4: Table S3. — Relative changes in the yeast metabolite abundance when cultured in succinate versus glucose. Differentially regulated metabolites observed during X. dendrorhous growth are shown. [file 12864_2015_1484_MOESM4_ESM.docx]

**Table S3. Relative changes in the yeast metabolite abundance when cultured in succinate versus glucose.**

| **Metabolite** | **Fold Change S/G^*^** | | | |  |  | **Fold Change S/G^*^** | | | |
| --- | --- | --- | --- | --- | --- | --- | --- | --- | --- | --- |
|  | **L** | **EE** | **EL** | **S** |  | **Metabolite** | **L** | **EE** | **EL** | **S** |
| **Sugar** |  |  |  |  |  | **Amino acid** | |  |  |  |
| Mannitol | **-11.42** | -10.39 | 1.69 | 1.10 |  | Phenylalanine | **-5.04** | -3.13 | -5.08 | **-3.93** |
| Sucrose | 3.91 | 1.31 | -20.81 | **-11.06** |  | Tyrosine | **-7.96** | -6.09 | -45.91 | **-35.68** |
| Fructose | **-6.43** | -8.10 | -18.20 | -19.05 |  | β-Alanine | -10.96 | -7.62 | -3.43 | **-4.44** |
| Arabinose | 1.01 | -7.72 | -1.07 | -1.77 |  | Histidine | 6.16 | 5.94 | -4.05 | **-3.56** |
| Xylose | -2.89 | -4.60 | -18.22 | **-7.06** |  | Glycine | -1.55 | -1.63 | 2.40 | **2.38** |
| Glucose | 2.15 | 1.99 | -18.12 | **-13.51** |  | Serine | **1.40** | 1.40 | -1.54 | -1.24 |
| Trehalose | -2.64 | -4.91 | 686.30 | **62.94** |  | Threonine | **1,19** | 1,24 | 1.56 | 1.57 |
| **Pentose Phosphate Pathway** | | |  |  |  | Cysteine | -1.19 | -1.36 | -40.99 | **-4.01** |
| Gluconate | **2.23** | 2.15 | 3.46 | 1.48 |  | Valine | -1.42 | -1.81 | 3.66 | **3.01** |
| Glycerate | **-8.10** | -6.05 | -1.42 | -1.16 |  | Leucine | **-1.24** | -1.17 | 1.05 | -1.09 |
| Gluconate-1,5-lactone | -1.28 | -1.58 | -3.10 | **-2.88** |  | Alanine | -1.02 | -1.31 | 4.22 | **2.91** |
| Erythrose-4-P | 4.90 | -1.19 | -15.84 | **-5.62** |  | Asparagine | **127.91** | **255.43** | 8.22 | 18.94 |
| Ribose | -4.43 | -4.98 | -2.29 | **-3.69** |  | Aspartate | 1.03 | **1.39** | 5.55 | **5.49** |
| **Glycolysis and Gluconeogenesis** | | | | |  | Lysine | -9.37 | -4,40 | -596,18 | -159.78 |
| Fructose-1-6-P | 7.38 | 5.19 | -2.03 | **-7.58** |  | Glutamine | **62.61** | 25.17 | 28.33 | 39.78 |
| Glycerate-3-P | **2.79** | 1.71 | 2.42 | **3.01** |  | Glutamate | 5.35 | **4.67** | 8.33 | **8.67** |
| Glicerate-2-P | 9.57 | 13.59 | 8.72 | 10.56 |  | Arginine | **-12.86** | -14.99 | 7.26 | **7.00** |
| PEP | **14.13** | 5.91 | 5.95 | **7.63** |  | Ornithine | **2.31** | 2.26 | 2.46 | 2.46 |
| Pyruvate | -1.10 | -1.13 | 3.08 | **2.89** |  | Pyroglutamate | 4.21 | 4.09 | 3.24 | 3.77 |
| **Tricarboxylic acid cycle** | | |  |  |  | **Nucleotide** |  |  |  |  |
| Citrate | **-1.98** | -1.83 | 3.57 | 2.40 |  | Adenosine | -2.01 | -2.48 | -6.28 | **-8.19** |
| Isocitrate | **17.26** | **22.76** | 3.83 | 2.72 |  | Adenine | **-5.03** | -3.81 | -2.20 | **-2.22** |
| a-Ketoglutarate | 1.56 | 1.76 | 4.70 | **4.54** |  | Thymine | **-3.48** | -4.80 | -30.93 | **-6.93** |
| Succinate | 6.40 | 6.95 | 13.43 | **8.68** |  | Uracil | **-3.25** | -2.51 | 3.49 | **5.39** |
| Fumarate | 2.40 | **2.73** | 6.16 | **6.64** |  |  |  |  |  |  |
| Malate | **2.43** | **2.47** | 7.91 | 7.84 |  |  |  |  |  |  |

*Mean fold changes in succinate compared with glucose. Statistical significance was estimated by *t*-test (p <0.02) which is shown as underlined values and the Benjamini-Hochberg (p <0.05) correction shown as bold values. L: lag phase, EE: Early exponential, EL: late exponential, S: Stationary, PEP: phosphoenolpyruvate.
